# Supplementary material for: Investigation of time to first presentation and extrahospital factors in the treatment of neovascular age-related macular degeneration: a retrospective cross-sectional study
Source: BMJ Open. 2017 Dec 10;7(12):e017771. doi: 10.1136/bmjopen-2017-017771 (PMC5778287; doi:10.1136/bmjopen-2017-017771)
Supplement: Supplementary file 1 [file bmjopen-2017-017771supp001.pdf]

Appendix 1: AMD Awareness Questionnaire.

No: Postcode:

AMD Awareness Questionnaire

Please circle the correct option

1. **Sex:** Male Female

2. **Age (years):**

3. **Educational Status:**
   1) Completed Primary School
   2) Completed Secondary School
   3) College Qualification
   4) University Degree
   5) Prefer not to say

4. **Employment status:**
   1) Unemployed
   2) Full time
   3) Part time
   4) Retired
   5) Other (specify):
   6) Prefer not to say

5. **Prior to now, have you ever been told you have Age Related Macular Degeneration (AMD)?**
   1) Yes
   2) No

IF 'Yes', MOVE ON TO QUESTION 7

6. **If no, have you ever heard of Age Related Macular Degeneration (AMD)?**
   1) Yes
   2) N

7. If you have heard of AMD, can you describe the condition and its symptoms?

____

____

8. Regardless of whether or not you are familiar with AMD, which of the following factors do you think increases the risk of developing AMD? (select all that apply)

1) Smoking
2) Vitamin deficiency
3) Age
4) Unprotected exposure to sunlight
5) Genetics
6) Sex

9. Do you currently smoke?

1) Yes
2) No

10. Is AMD a treatable condition?

1) Yes
2) No

11. If yes, do you know what treatments are available?

____

____

12. If you were worried about your eyesight, where would you go for advice?

1) Ophthalmologist
2) GP
3) Pharmacist
4) Optician
5) Other (specify):
