# Supplementary material for: Investigation of time to first presentation and extrahospital factors in the treatment of neovascular age-related macular degeneration: a retrospective cross-sectional study
Source: BMJ Open. 2017 Dec 10;7(12):e017771. doi: 10.1136/bmjopen-2017-017771 (PMC5778287; doi:10.1136/bmjopen-2017-017771)
Supplement: Supplementary file 2 [file bmjopen-2017-017771supp002.pdf]

Appendix 2: Power calculation for AMD awareness survey sample size.

Sample Size:X-Sectional, Cohort, & Randomized Clinical Trials

Two-sided significance level(1-alpha):	95
Power(1-beta, % chance of detecting):	80
Ratio of sample size, Unexposed/Exposed:	1
Percent of Exposed with Outcome:	25
Odds Ratio:	6.3
Risk/Prevalence Ratio:	5
Risk/Prevalence difference:	20

	Kelsey	Fleiss	Fleiss with CC
Sample Size - Exposed	51	49	59
Sample Size-Nonexposed	51	49	59
Total sample size:	102	98	118

References
Kelsey et al., Methods in Observational Epidemiology 2nd Edition, Table 12-15
Fleiss, Statistical Methods for Rates and Proportions, formulas 3.18 &3.19
CC = continuity correction

Based on a global survey investigating AMD awareness by AMD Alliance International, the level of awareness in the UK was 16% in 2005.[38] Allowing for increase in awareness over time (demonstrated by studies in other countries), hence assuming a slightly higher level of awareness (~25%) in south-east Scotland, we would have a power of 80% to detect this with a total sample size of 118 patients.

Appendix 3: Table comparing summary demographic data for included vs excluded case notes.

Patient demographics	Analysed (*n* = 195)	Excluded (*n* = 120)
Sex (% female)	61.5	58.3
Mean age (years)	77.7	78.4
Percentage of patients presenting with first affected eye	95.9%	97.5%

Appendix 4: Table showing breakdown of case notes excluded from study.

**Total case notes identified**	**315**
Co-existence of ocular comorbidities that give rise to choroidal neovascularization	23
Symptom duration not recorded	76
Lost to follow-up	21
**Case notes included in study**	**195**
