# Supplementary material for: Investigation of time to first presentation and extrahospital factors in the treatment of neovascular age-related macular degeneration: a retrospective cross-sectional study
Source: BMJ Open. 2017 Dec 10;7(12):e017771. doi: 10.1136/bmjopen-2017-017771 (PMC5778287; doi:10.1136/bmjopen-2017-017771)
Supplement: Author's manuscript [file bmjopen-2017-017771.draft_revisions.pdf]

BMJ Open is committed to open peer review. As part of this commitment we make the peer review history of every article we publish publicly available.

When an article is published we post the peer reviewers' comments and the authors' responses online. We also post the versions of the paper that were used during peer review. These are the versions that the peer review comments apply to.

The versions of the paper that follow are the versions that were submitted during the peer review process. They are not the versions of record or the final published versions. They should not be cited or distributed as the published version of this manuscript.

BMJ Open is an open access journal and the full, final, typeset and author-corrected version of record of the manuscript is available on our site with no access controls, subscription charges or pay-per-view fees (http://bmjopen.bmj.com).

If you have any questions on BMJ Open's open peer review process please email editorial.bmjopen@bmj.com

BMJ Open

Time to first presentation and extra-hospital factors contribute to delays in the treatment of wet age-related macular degeneration (AMD): a retrospective cross-sectional study

Journal:	*BMJ Open*
Manuscript ID	bmjopen-2017-017771
Article Type:	Research
Date Submitted by the Author:	16-May-2017
Complete List of Authors:	Sim, Peng Yong; The University of Edinburgh<br>Gajree, Sonul; Gartnavel General Hospital<br>Dhillon, Baljean; The University of Edinburgh; Princess Alexandra Eye Pavilion<br>Borooah, Shyamanga; Moorfields Eye Hospital
**Primary Subject Heading**:	Ophthalmology
Secondary Subject Heading:	Public health
Keywords:	OPHTHALMOLOGY, Medical retina < OPHTHALMOLOGY, PUBLIC HEALTH

SCHOLARONE™
Manuscripts

For peer review only

Time to first presentation and extra-hospital factors contribute to delays in the treatment of wet age-related macular degeneration (AMD): a retrospective cross-sectional study

Peng Yong Sim[1]
Sonul Gajree[2]
Baljean Dhillon[1,3]
Shyamanga Borooah[4]

[1]The University of Edinburgh, Edinburgh, UK
[2]Gartnavel General Hospital, Glasgow, UK
[3]The Princess Alexandra Eye Pavilion, Edinburgh, UK
[4]Moorfields Eye Hospital, London, UK

Corresponding author:
Name : Peng Yong Sim
Email : pengyong91@gmail.com

Conflict of interest: None

For peer review only

Abstract

Objectives: To assess the time from symptom onset to treatment for wet age-related macular degeneration (AMD) and to measure the awareness of AMD in south-east Scotland.

Design: Retrospective cross-sectional study.

Setting: Secondary care, south-east of Scotland.

Methods: Patients treated with intravitreal therapy (IVT) for wet AMD in south-east Scotland between 2013–2015 were identified using a treatment register. Notes were retrospectively reviewed. We measured time from A) symptom onset to first presentation at primary care, B) referral to ophthalmic clinic appointment and C) ophthalmic clinic appointment to first IVT treatment. To investigate AMD awareness, we performed a cluster random sample survey of patients visiting non-AMD ophthalmic clinics using a previously validated 12-item questionnaire.

Results: 195 patients (mean age 78) were included in the study. The mean delays between the different stages – A, B and C – were 54.2 (95% CI±13), 28.2 (95% CI±4.0) and 31.5 (95% CI±3.6) days respectively. There was an additional mean delay of 7.5 (95% CI±1.6) days when patients were indirectly referred by optometrists via general practitioners ($p<0.05$). 140 patients (mean age 78) participated in the awareness survey; 62.1% reported being "aware" of AMD but only 37.3% described AMD symptoms correctly.

Conclusions: There is significant delay at every step of the wet AMD patient pathway in south-east Scotland. Our findings suggest that suboptimal awareness of AMD symptoms could

For peer review only - http://bmjopen.bmj.com/site/about/guidelines.xhtml

account for a substantial delay in presentation from symptom onset. This highlights the need to increase awareness of AMD which may optimise visual outcomes for patients.

Article Summary (Strengths and Limitations of this Study)

- Large sample size of patients.
- Case notes of consecutive patients identified systematically using a treatment clinic register over 2 years.
- Demographic factors such as age, gender, education, social class and smoking status were taken into account for analyses of AMD awareness.
- Unable to ascertain direct association between low disease awareness and delay in treatment for patients with wet AMD as different cohort of patients were examined.
- Retrospective analysis of notes from disease register.

For peer review only

For peer review only - http://bmjopen.bmj.com/site/about/guidelines.xhtml

Introduction

Age-related macular degeneration (AMD) is the leading cause of vision loss in the developed world.[1] There are two main forms of AMD. The first is dry AMD which accounts for the majority of AMD cases and results from the deposition of materials deep to the retina which eventually leads to the slow degeneration of retinal cells resulting in blindness. Wet AMD accounts for the remaining cases of AMD and results from the development of new blood vessels deep to the retina which leak or bleed resulting in symptoms of new distortion or vision loss. Wet AMD results in irreversible blindness if left untreated and accounts for 90% of the cases of blind registration resulting from AMD.[2] The main risk factors associated with the AMD are age and smoking.[3] Cases of blindness resulting from AMD are predicted to increase together with an ageing population.[4]

An effective treatment is currently available to preserve vision in wet AMD in the form of intravitreal therapy (IVT) with anti-vascular endothelial growth factor agents.[5–8] They have been shown to be effective in maintaining long-term vision in the majority of patients affected by wet AMD.[9] Delay in instituting IVT treatment in new cases of wet AMD has been shown to be one of the most important factors negatively impacting final visual outcome.[10,11] Consequently, the early diagnosis and treatment is crucial to not only improving visual outcomes in AMD, but also to reduce the social and economic burden of blindness resulting from wet macular degeneration.[12,13]

Delays from symptom onset to treatment can be experienced at different stages of the patient care pathway for new onset wet AMD. These include: 1) time of first symptom onset to presentation at primary care practitioner, 2) time from primary care referral to presentation at ophthalmic clinic and 3) time from ophthalmic clinic to first IVT treatment (Fig. 1). These early

For peer review only

stages of the care pathway also represent the periods during which lesions may be most active and amenable to the benefits of therapy.[14]

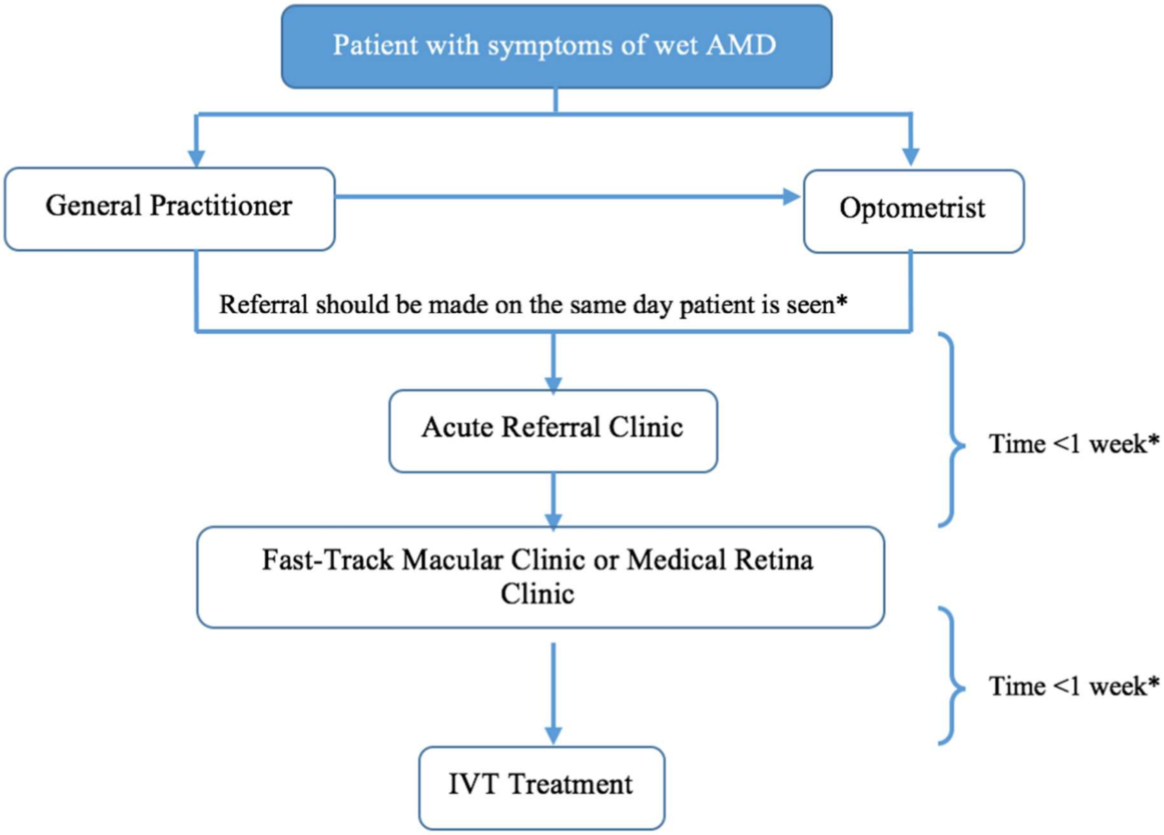

Fig 1. Flow chart depicting the typical care pathway of a patient with wet AMD in south-east Scotland. *Based on the recommendations by the Royal College of Ophthalmologists in its 2013 AMD guideline.[15]

There have been many published reports investigating intra-hospital factors such as the time from first ophthalmic clinic visit to first IVT treatment.[10,11,16] However, there is a scarcity of literature reporting the extra-hospital factors such as the time from symptom onset to presentation at ophthalmic clinic. In addition, despite its significance in causing blindness, limited research has been performed to investigate AMD awareness. An exploration of patient's awareness and knowledge of disease has been demonstrated in other chronic diseases such as stroke and cancer[17,18], with increased awareness associated with improved patient outcomes.[19,20]

For peer review only - http://bmjopen.bmj.com/site/about/guidelines.xhtml

The primary objectives of this study were twofold; first, to assess the time between the different stages of the AMD care pathway in patients treated in south-east Scotland and second, to evaluate patients' awareness of AMD, its risk factors and treatment options.

Methods

Case notes of consecutive patients diagnosed and treated with IVT for wet AMD in NHS Lothian since September 2013 were identified using a treatment clinic register. A 2013 cut-off point was chosen to reflect the updated guidelines on AMD by the Royal College of Ophthalmologists (RCOphth) which were published at the time.[15] The guidelines recommended that all patients with suspected AMD should be seen by a retinal specialist within one week of referral, and that treatment should commence within one week of first ophthalmic appointment (Fig. 1).

In this study, the main outcome measures were 1) time from symptom onset to first presentation at primary care (i.e. duration of visual symptoms before initial presentation), 2) time from primary care referral to ophthalmic clinic appointment, and 3) time from ophthalmic clinic appointment to first IVT treatment. A total of 315 case notes were identified; 120 of the 315 were excluded due to incomplete data and the co-existence of ocular comorbidities that gave rise to choroidal neovascularisation. This study was accepted and approved by the NHS Lothian quality improvement team.

In order to investigate patients' awareness of AMD, a cluster random sample of patients visiting ophthalmic clinics for non-AMD disease in NHS Lothian was surveyed using a 12-item questionnaire (see Supplementary File). Questions were adapted from a previously validated questionnaire[21] and served to ascertain each patient's knowledge of AMD and its risk factors. Patients were asked for their demographic details, including age, sex, education and postcode

For peer review only - http://bmjopen.bmj.com/site/about/guidelines.xhtml

of residence. Socioeconomic deprivation scores (social class) were calculated for all patients from postcode data at the time of interview using the Scottish Index of Multiple Deprivation (SIMD).[22] The SIMD combines weighted data on seven domains (income, employment, education, housing, health, crime and geographical access) and is officially sanctioned by the Scottish Government as a measure of multiple deprivation.[23,24]

The first part of the questionnaire explored patients' familiarity with AMD and its risk factors. The second part enquired about patients' smoking status and their awareness of available treatments for AMD. Surveys were distributed and collected by the same researcher, who remained nearby to answer any questions about instructions. No additional assistance was provided. Data for the survey was collected from November 18 to 31, 2015. The data was analysed using Pearson χ^2 tests except for education and social class where χ^2 tests for trend were performed. Data analysis was done using IBM SPSS Statistics for Windows, version 23 (IBM Corp., Armonk, N.Y., USA).

Results

Delay in presentation, referral and treatment of AMD in south-east Scotland

195 case notes were analysed in total. 120 (61.5%) patients were female, with a mean age of 78 years. Nearly all patients (187; 95.9%) presented with wet AMD affecting the first eye. The overall mean time from symptom onset to presentation was 54.2 (95% CI±13) days. As for referrals to ophthalmology, 118 (60.5%) of these were direct from optometrists, 5 (2.6%) were direct from GPs, and 52 (26.7%) were made by optometrists via GPs. The remaining referrals were from other hospitals, other ophthalmology clinics, and screening programmes.

For peer review only - http://bmjopen.bmj.com/site/about/guidelines.xhtml

The mean time from referral to ophthalmic clinic appointment was 28.2 days (95% CI±4.0 days). There was a significant additional mean delay of 7.5 ($p<0.05$) (95 %CI±1.6) days when patients were referred from their optometrist via their GP. During clinic appointments, fundus fluorescein angiogram was performed in approximately one third of patients (66/195). The mean time from clinic to first IVT treatment was 31.5 (95% CI±3.6) days (Fig. 2).

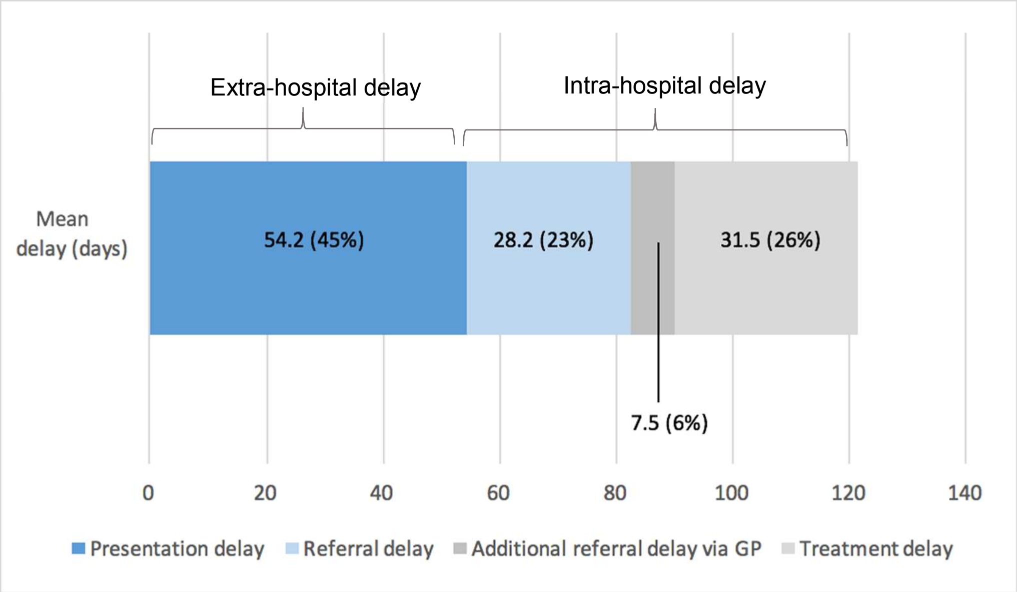

Fig 2. Breakdown of the total delay (121.4 days) from symptom onset to treatment for patients with new wet AMD in south-east Scotland.

Awareness of AMD and its risk factors

The delay from symptom onset to first injection resulted from both intra-hospital and extra-hospital factors. We have already identified that when optometrists referred via the GPs instead of directly to the hospital eye service this resulted in a significant increased delay. However, even this delay is overshadowed by the mean delay from symptom onset to presentation at primary care service. In order to better understand patient factors that may have resulted in this delay in presentation we performed a questionnaire survey on patients with unrelated disease in the eye service. A total of 142 patients were approached in non-AMD ophthalmic clinics. These

For peer review only - http://bmjopen.bmj.com/site/about/guidelines.xhtml

clinics included glaucoma, ocular motility and general outpatient clinics. 140 patients agreed to participate. Two refused because of unwillingness and inability to understand the purpose of the questionnaire due to deafness respectively.

The cohort included 61 (43.6%) male and 79 (56.4%) female with a median age of 73 (range 17-93), comprising all social classes. The education level of patients ranged from primary education to university degree. Details of the demographic data are given in Table 1.

Table 1. Demographic data of patients (n=140)

**Variable**	***n***	**%**
Gender		
Male	61	43.6
Female	79	56.4
Age (years)		
<50	19	13.6
≥50	121	86.4
Highest education level attained		
Primary school	4	2.9%
Secondary school	78	55.7%
College	31	22.1%
University degree	27	19.3%
Social class		
I	13	9.3%
II	25	17.8%
III	20	14.3%
IV	27	19.3%
V	55	39.3%
Smoking status		
Current smoker	11	7.9%
Ex- or non-smoker	129	92.1%

Of the 140 respondents, 87 (62.1%) reported being “aware” of AMD. 14 (10%) had previously been diagnosed with AMD. 10 of these 14 patients (71.4%) were able to provide a correct description of the symptoms of AMD. For those patients without a prior diagnosis, only 47/126 (37.3%) were able to correctly report the symptoms of AMD. There was a significant difference when comparing the responses of those who had a previous diagnosis of AMD to those without AMD (p=0.013). Overall female respondents were more likely than male respondents to report

For peer review only - http://bmjopen.bmj.com/site/about/guidelines.xhtml

awareness of AMD (p=0.015) (Table 2). Increased awareness of AMD was also seen with higher levels of education (p=0.001).

Table 2. Respondents indicating awareness of AMD (n=140)

Characteristic[a]	No. Indicating Awareness / Total No. (%)
Gender distribution	
Male	31/61 (50.8)
Female	56/79 (70.9)
p value	0.015
Age (years)	
<50	8/19 (42.1)
≥50	79/121 (65.3)
p value	0.053
Highest education level attained	
Primary school	4/7 (57.1)
Secondary school	37/75 (49.3)
College	23/31 (74.2)
University degree	23/27 (85.2)
p value[b]	0.001
Social class	
I	8/13 (61.5)
II	18/25 (72.0)
III	9/20 (45.0)
IV	21/27 (77.8)
V	31/55 (56.4)
p value[b]	0.537
Smoking status	
Current smoker	6/11 (54.5)
Ex- or non-smoker	81/129 (62.8)
p value	0.588

[a] Unless otherwise indicated, *p* values are derived using the Pearson χ^2 test.
[b] Derived using the χ^2 for trend.

The top risk factor for AMD correctly considered by patients was age (127/140 - 90.7%). The other risk factors identified included smoking in 82 (58.6%), unprotected UV exposure in 62 (44.3%), genetic predisposition in 62 (44.3%), vitamin deficiency in 54 (38.6%) and gender in 15 (10.7%).

87 (62.1%) of patients thought that AMD was a treatable condition. However, only 20/87 (23%) were able to provide correct information on the available treatments (i.e. eye injections and laser therapy). The majority of patients (91/140, 65%) considered opticians to be their first port of call if they had vision problems. Other healthcare professionals cited as first port of call included

For peer review only - http://bmjopen.bmj.com/site/about/guidelines.xhtml

general practitioners in 28 (20%) and ophthalmologists in 21 (15%).

Discussion

The Royal College of Ophthalmologists (RCOphth) has recently updated its guidance on suggested waiting times for IVT treatment in wet AMD in the hospital setting. It recommends that all patients should be seen by a retinal specialist within one week of primary care referral, and should begin treatment within one week following this.[15] The new guidelines place increased importance on correct diagnosis and urgent referral from primary care and place increasing emphasis on hospital eye services to provide capacity for urgent new AMD cases in addition to the treatment of existing wet AMD patients. However, this study finds that there are significant delays at each step of the wet AMD care pathway in south-east Scotland; both the waiting times from 1) primary care referral to ophthalmic clinic and 2) initial ophthalmic assessment to treatment are about four times as long as the recommended gold standard. In addition, there is a further one-week delay on average when indirect referrals are made by optometrists via GP. Similar findings have also been reported in previous studies which have demonstrated similar, if not longer, delays for intra-hospital pathways (i.e. from initial ophthalmic assessment to treatment).[10,11,16]

To our knowledge, this is the first study to evaluate the time from symptom onset to presentation at clinic (extra-hospital pathway) for patients with wet AMD in the UK. Our findings demonstrate that this not only represents a major source of delay but also accounts for the greatest proportion of the delay in the wet AMD care pathway in south-east Scotland. This represents an important target for improvement to reduce vision loss resulting from delay in the wet AMD care pathway.[25] This delay is likely to be complex and multifactorial, involving patients, eye care providers and healthcare systems. Barriers to early presentation might include a lack of

For peer review only - http://bmjopen.bmj.com/site/about/guidelines.xhtml

awareness of wet AMD among patients, self-examination by patients and screening of the disease by non-retina specialists.

At present, the diagnosis of new wet AMD, especially for the first affected eye, still very much relies on self-recognition of visual symptoms by patient themselves. This is however problematic as those affected in only one eye tend not to be aware of the visual change and may therefore remain "asymptomatic" for a considerable length of time.[26] Indeed, this seemed to be case in our study in which nearly all patients presented with wet AMD affecting the first eye.

There is evidence to show that the best correct visual acuity at the time of diagnosis of wet AMD is worse for the first affected eye when compared to that of the second eye.[27] In addition, previous studies have shown that the visual prognosis of the first affected eye following one year of treatment is usually worse compared to that of the second affected eye in wet AMD.[28,29] These better outcomes of the second affected eye are most likely due to increased awareness and more frequent monitoring of the second eye as part of a systematic bilateral follow-up examination for the first affected eye. These factors would seemingly translate into a shorter delay in presentation for the second affected eye but it is should be noted that this association was not explored in our study and remains to be investigated. Nonetheless, the considerable delay in presentation for the first affected eye demonstrated in our study highlights the importance of early detection and treatment.

From the patient's perspective, the delay in symptom recognition can be addressed to a certain extent by self-examination. Patients, especially those with an increased risk of developing wet AMD, should be educated and made aware of symptoms such as new visual distortion and sudden reduction in vision. This can be achieved by encouraging patients to use suitable spaced self-tests of vision which examine one eye at a time to prevent compensation from the

For peer review only - http://bmjopen.bmj.com/site/about/guidelines.xhtml

good eye. The standard Amsler test has long been recommended as the standard self-monitoring test but there has been increasing reservation about its utility as a diagnostic tool due to its insufficient reliability and variable sensitivity.[30,31] The advent of more innovative, cost saving technologies may circumvent these issues and make implementation of self-examination on a wider public scale more feasible in the near future.[32,33]

In this study, we chose to investigate patients' awareness of AMD because it is clear that a lack of disease awareness is a common factor for delayed presentation in other eye conditions such as glaucoma, retinal detachment and central retinal artery occlusion.[34,35] The only previous study to investigate AMD awareness in the UK population showed a low awareness (16%).[36] Our study adds to the existing literature by demonstrating that public awareness of AMD is still limited. Our survey shows that awareness of AMD is unacceptably low (37%), especially considering that this condition is the leading cause of blindness in developed countries.[1] The low awareness of AMD is also consistent with the low levels of awareness of AMD in other countries.[21,36,38–43] It is likely that our findings underestimate the true scale of awareness among the general population because we sampled ophthalmic patients who, by virtue of being surveyed in an eye hospital, are presumably somewhat more attuned to common eye diseases.

These findings are important given the severity of the consequences of delayed presentation in AMD and the ready availability of an effective treatment to prevent visual loss. We identified AMD-naive male patients and those with lower education levels to have a particularly low awareness of warning symptoms of AMD, suggesting the need for targeted intervention for these subgroups. As increased awareness can lead patients to seek appropriate medical care, improving awareness would logically lead to better visual prognoses for patients.

For peer review only

There is currently still a need for a unified national awareness campaign on AMD in the UK. A recent report by the Royal National Institute of Blind People highlighted that most initiatives at improving AMD awareness in the UK still operate at a local level.[44] Even then, these efforts often comprised of educational talks targeted at existing patients, rather than raising public awareness. The need for a national campaign has also been recognised by the Macular Society which has made increasing AMD awareness one of the main objectives of its five-year national strategy.[45]

Although some progress has been made since,[46] there is still room for improvement. Current awareness interventions need to be further optimised for a sustained impact. A promising step would be the adoption of the multi-layered approach as adopted by other developed countries.[47] This approach saw the use of a campaign which included a diverse range of activities such as promoting education programmes for patients and primary care, running a national advertising campaign and providing free mobile screening. The end of this focused campaign saw a dramatic increase in AMD awareness and the number of the population requesting fundus examination for symptoms of AMD.[47] The implementation of a similar public health strategy in the UK may achieve similar desirable effects but further research is needed to evaluate the effectiveness of this approach in the UK population. Another important gap highlighted by our study is the underappreciated link between smoking and AMD. This represents a potent novel health promotional tool and awareness could be increased by incorporating information in existing campaigns with other smoking related diseases.[48,49]

Conclusion

There is significant delay at every step of the care pathway for patients with wet AMD in south-east Scotland. We also show that awareness and knowledge of AMD are suboptimal. This lack

For peer review only - http://bmjopen.bmj.com/site/about/guidelines.xhtml

of AMD could account for the long presentation delay of AMD to primary care. This suggests that efforts to educate the public regarding AMD may lead to earlier presentation and hence improved visual outcomes in patients.

Acknowledgement

None

Funding

SB was sponsored by the Wellcome Trust Scottish Translational Medicine and Therapeutics Initiative (STMTI) scheme (grant number R42141) during the course of this research.

Competing interests

None declared.

Contributors

BD and SB were involved in conception and design of the study. PS and SG were involved in data acquisition, analysis and interpretation. PS was involved in first draft of manuscript. SG, SB and BD were involved in revising and critically appraising manuscript. PS, SG, BD and SB were involved in final approval for publication. BD and SB are guarantors.

For peer review only

For peer review only - http://bmjopen.bmj.com/site/about/guidelines.xhtml

References

1. World Health Organisation. Priority eye diseases. 2017. Available at: http://www.who.int/blindness/causes/priority/en/index7.html [Accessed January 19, 2017].

2. The Royal College of Ophthalmologists. Commissioning Contemporary AMD Services: A guide for commissioners and clinicians. 2007.

3. Smith W, Assink J, Klein R, et al. Risk factors for age-related macular degeneration: Pooled findings from three continents. Ophthalmology. 2001; 108(4): 697–704.

4. Minassian DC, Reidy A, Lightstone A, et al. Modelling the prevalence of age-related macular degeneration (2010-2020) in the UK: expected impact of anti-vascular endothelial growth factor (VEGF) therapy. Br. J. Ophthalmol. 2011; 95(10): 1433–1436.

5. Brown DM, Kaiser PK, Michels M, et al. Ranibizumab versus verteporfin for neovascular age-related macular degeneration. N. Engl. J. Med. 2006; 355(14): 1432–44.

6. Brown DM, Michels M, Kaiser PK, et al. Ranibizumab versus Verteporfin Photodynamic Therapy for Neovascular Age-Related Macular Degeneration: Two-Year Results of the ANCHOR Study. Ophthalmology. 2009; 116(1): 57–65.e5.

7. Busbee BG, Ho AC, Brown DM, et al. Twelve-Month Efficacy and Safety of 0 . 5 mg or 2 . 0 mg Ranibizumab in Patients with Subfoveal Neovascular Age-related Macular. Ophthalmology. 2013: 1046–1056.

8. Heier JS, Brown DM, Chong V, et al. Intravitreal aflibercept (VEGF trap-eye) in wet age-related macular degeneration. Ophthalmology. 2012; 119(12): 2537–2548.

9. Rofagha S, Bhisitkul RB, Boyer DS, et al. Seven-year outcomes in ranibizumab-treated patients in ANCHOR, MARINA, and HORIZON: A multicenter cohort study (SEVEN-UP). Ophthalmology. 2013; 120(11): 2292–2299.

10. Lim JH, Wickremasinghe SS, Xie J, et al. Delay to treatment and visual outcomes in patients treated with anti-vascular endothelial growth factor for age-related macular degeneration. Am. J.

For peer review only

For peer review only - http://bmjopen.bmj.com/site/about/guidelines.xhtml

Ophthalmol. 2012; 153(4): 678–686.e2.

11. Takahashi H, Ohkubo Y, Sato A, et al. Relationship Between Visual Prognosis and Delay of Intravitreal Injection of Ranibizumab When Treating Age-Related Macular Degeneration. Retina. 2015; 35(7): 1331–1338.

12. Bonastre J, Le Pen C, Soubrane G, et al. The burden of age-related macular degeneration: results of a cohort study in two French referral centres. Pharmacoeconomics. 2003; 21(3): 181–190.

13. Hopley C, Salkeld G, Wang JJ, et al. Cost utility of screening and treatment for early age related macular degeneration with zinc and antioxidants. Br. J. Ophthalmol. 2004; 88(4): 450–454.

14. Gonzales CR. Enhanced efficacy associated with early treatment of neovascular age-related macular degeneration with pegaptanib sodium: an exploratory analysis. Retina. 2005; 25(7): 815–27.

15. Chakravarthy U, Williams M, Amoaku W, et al. The Royal College of Ophthalmologists Guidelines on AMD: Executive Summary. Eye. 2013; 27(12): 1429–1431.

16. Arias L, Armadá F, Donate J, et al. Delay in treating age-related macular degeneration in Spain is associated with progressive vision loss. Eye. 2009; 23(2): 326–33.

17. Garraway WM, Whisnant JP. The changing pattern of hypertension and the declining incidence of stroke. JAMA. 1987; 258(2): 214–217.

18. Worden JK, Solomon LJ, Flynn BS, et al. A community-wide program in breast self-examination training and maintenance. Prev. Med. (Baltim). 1990; 19(3): 254–269.

19. Kim YS, Park SS, Bae HJ, et al. Stroke awareness decreases prehospital delay after acute ischemic stroke in Korea. BMC Neurol. 2011; 11: 2.

20. Niksic M, Rachet B, Duffy SW, et al. Is cancer survival associated with cancer symptom awareness and barriers to seeking medical help in England? An ecological study. Br. J. Cancer. 2016; 115(7): 876–886.

For peer review only

21. Livingston PM, McCarty CA, Taylor HR. Knowledge, attitudes, and self care practices associated with age related eye disease in Australia. Br. J. Ophthalmol. 1998; 82(7): 780–785.

22. The Scottish Government. Scottish Index of Multiple Deprivation 2012: A National Statistics Publication for Scotland; 2012. Available at: http://simd.scotland.gov.uk/publication-2012/

23. Aitken E, Dempster N, Ceresa C, et al. The impact of socioeconomic deprivation on outcomes following renal transplantation in the West of Scotland. Transplant. Proc. 2013; 45(6): 2176–2183.

24. Low L, Law JP, Hodson J, et al. Impact of socioeconomic deprivation on the development of diabetic retinopathy: a population-based, cross-sectional and longitudinal study over 12 years. BMJ Open. 2015; 5(4): e007290.

25. Rasmussen A, Brandi S, Fuchs J, et al. Visual outcomes in relation to time to treatment in neovascular age-related macular degeneration. Acta Ophthalmol. 2015; 93(7): 616–620.

26. Bressler NM. Early detection and treatment of neovascular age-related macular degeneration. J. Am. Board Fam. Pract. 2002; 15(2): 142–52.

27. Chevreaud O, Semoun O, Blanco-Garavito R, et al. Visual acuity at presentation in the second eye versus first eye in patients with exudative age-related macular degeneration. Eur. J. Ophthalmol. 2015; 26(1): 44–47.

28. Muniraju R, Jayashree R, Sivaprasad S. Outcomes of Second eye Versus First eye in Patients Treated with Ranibizumab for Bilateral Neovascular Age Related Macular Degeneration. Invest. Ophthalmol. Vis. Sci. 2013; 54(15): 2013.

29. Chew J, Broadhead G, Li H, et al. Visual outcomes of ranibizumab treatment in fellow eyes of exudative AMD. Invest. Ophthalmol. Vis. Sci. 2013; 54(15): 3832.

30. Zaidi FH, Cheong-Leen R, Gair EJ, et al. The Amsler chart is of doubtful value in retinal screening for early laser therapy of subretinal membranes. The West London Survey. Eye (Lond). 2004; 18(5): 503–8.

31. Faes L, Bodmer NS, Bachmann LM, et al. Diagnostic accuracy of the Amsler grid and the

For peer review only

For peer review only - http://bmjopen.bmj.com/site/about/guidelines.xhtml

preferential hyperacuity perimetry in the screening of patients with age-related macular degeneration: systematic review and meta-analysis. Eye (Lond). 2014; 28(7): 788–96.

32. Winther C, Frisén L. Self-Testing of Vision in Age-Related Macula Degeneration: A Longitudinal Pilot Study Using a Smartphone-Based Rarebit Test, Self-Testing of Vision in Age-Related Macula Degeneration: A Longitudinal Pilot Study Using a Smartphone-Based Rarebit Test. J. Ophthalmol. J. Ophthalmol. 2015; 2015, 2015: e285463.

33. Schwartz R, Loewenstein A. Early detection of age related macular degeneration: current status. Int. J. Retin. Vitr. 2015; 1(1): 1–8.

34. Saw S-M, Gazzard G, Friedman D, et al. Awareness of glaucoma, and health beliefs of patients suffering primary acute angle closure. Br. J. Ophthalmol. 2003; 87(4): 446–449.

35. Uhr JH, Mishra K, Wei C, et al. Awareness and knowledge of emergent ophthalmic disease among patients in an internal medicine clinic. JAMA Ophthalmol. 2016; 134(4): 424–431.

36. AMD Alliance International. Awareness of age-related macular degeneration and associated risk factors. AMD Global Report. 2005; Available at: http://www.eyeresearch.org/pdf/AMD_Report_Aug22_1046.pdf

37. Taylor HR, Keeffe JE. World blindness: a 21st century perspective. Br. J. Ophthalmol. 2001; 85(3): 261–6.

38. Lau J, Lee V, Fan D, et al. Knowledge about cataract, glaucoma, and age related macular degeneration in the Hong Kong Chinese population. Br. J. Ophthalmol. 2002; 86: 1080–1084.

39. Sanjay S, Neo HY, Sangtam T, et al. Survey on the knowledge of age-related macular degeneration and its risk factors among Singapore residents. Clin. Exp. Ophthalmol. 2009; 37(8): 795–800.

40. Thapa R, Bajimaya S, Paudyal G, et al. Population awareness of diabetic eye disease and age related macular degeneration in Nepal: the Bhaktapur Retina Study. BMC Ophthalmol. 2015; 15(1): 188.

41. Islam FMA, Chakrabarti R, Islam SZ, et al. Factors associated with awareness, attitudes and

For peer review only

practices regarding common eye diseases in the general population in a rural district in Bangladesh: The Bangladesh population-based diabetes and eye study (BPDES). PLoS One. 2015; 10(7): 1–12.

42. Scott AW, Bressler NM, Ffolkes S, et al. Public Attitudes About Eye and Vision Health. JAMA Ophthalmol. 2016; 21287(10): 1–8.

43. Zhang C, Zhang G, Ma N, et al. Awareness of Age-related Macular Degeneration and Its Risk Factors among Beijing Residents in China. 2017; 130(2): 155–159.

44. Royal National Institute of Blind People. Don't lose sight! Don't delay! Perspectives on the wet age-related macular degeneration (wet AMD) patient journey. RNIB Campaign Report. 2013. Available at: https://www.rnib.org.uk/sites/default/files/Don%27t%20lose%20sight%20don%27t%20delay%20Campaign%20report.pdf [Accessed April 15, 2017]

45. Macular Society. Strategy 2016-2020. 2016. Available at: https://www.macularsociety.org/sites/default/files/Macular%20Society%20strategy%202016-2020.pdf [Accessed April 15, 2017]

46. Royal National Insitute of Blind People. Charity announces new awareness week for macular conditions. 2015. Available at: http://www.rnib.org.uk/charity-announces-new-awareness-week-macular-conditions [Accessed April 15, 2017]

47. Heraghty J, Cummins R. A layered approach to raising public awareness of Macular Degeneration in Australia. Am. J. Public Health. 2012; 102(9): 1655–1659.

48. Thornton J, Edwards R, Harrison RA, et al. 'Smoke gets in your eyes': A research-informed professional education and advocacy programme. J. Public Health (Bangkok). 2007; 29(2): 142–146.

49. Thompson C, Harrison RA, Wilkinson SC, et al. Attitudes of community optometrists to smoking cessation: An untapped opportunity overlooked? Ophthalmic Physiol. Opt. 2007; 27(4): 389–393.

For peer review only

For peer review only - http://bmjopen.bmj.com/site/about/guidelines.xhtml

For peer review only

For peer review only - http://bmjopen.bmj.com/site/about/guidelines.xhtml

No: **Postcode**:

AMD Awareness Questionnaire

Please circle the correct option

1. **Sex:** Male Female

2. **Age (years):**

3. **Educational Status:**
   1) Completed Primary School
   2) Completed Secondary School
   3) College Qualification
   4) University Degree
   5) Prefer not to say

4. **Employment status:**
   1) Unemployed
   2) Full time
   3) Part time
   4) Retired
   5) Other (specify):
   6) Prefer not to say

5. **Prior to now, have you ever been told you have Age Related Macular Degeneration (AMD)?**
   1) Yes
   2) No

IF 'Yes', MOVE ON TO QUESTION 7

6. **If no, have you ever heard of Age Related Macular Degeneration (AMD)?**
   1) Yes
   2) No

For peer review only

7. If you have heard of AMD, can you describe the condition and its symptoms?

____

____

8. Regardless of whether or not you are familiar with AMD, which of the following factors do you think increases the risk of developing AMD? (select all that apply)

1) Smoking
2) Vitamin deficiency
3) Age
4) Unprotected exposure to sunlight
5) Genetics
6) Sex

9. Do you currently smoke?

1) Yes
2) No

10. Is AMD a treatable condition?

1) Yes
2) No

11. If yes, do you know what treatments are available?

____

____

12. If you were worried about your eyesight, where would you go for advice?

1) Ophthalmologist
2) GP
3) Pharmacist
4) Optician
5) Other (specify):

For peer review only

STROBE 2007 (v4) Statement—Checklist of items that should be included in reports of *cross-sectional studies*

Section/Topic	Item #	Recommendation	Reported on page #
**Title and abstract**	1	(*a*) Indicate the study's design with a commonly used term in the title or the abstract	1
		(*b*) Provide in the abstract an informative and balanced summary of what was done and what was found	2
**Introduction**			
Background/rationale	2	Explain the scientific background and rationale for the investigation being reported	4-5
Objectives	3	State specific objectives, including any prespecified hypotheses	6
**Methods**			
Study design	4	Present key elements of study design early in the paper	6
Setting	5	Describe the setting, locations, and relevant dates, including periods of recruitment, exposure, follow-up, and data collection	6
Participants	6	(*a*) Give the eligibility criteria, and the sources and methods of selection of participants	6
Variables	7	Clearly define all outcomes, exposures, predictors, potential confounders, and effect modifiers. Give diagnostic criteria, if applicable	6
Data sources/ measurement	8*	For each variable of interest, give sources of data and details of methods of assessment (measurement). Describe comparability of assessment methods if there is more than one group	6
Bias	9	Describe any efforts to address potential sources of bias	6-7
Study size	10	Explain how the study size was arrived at	6-7
Quantitative variables	11	Explain how quantitative variables were handled in the analyses. If applicable, describe which groupings were chosen and why	6-7
Statistical methods	12	(*a*) Describe all statistical methods, including those used to control for confounding	6-7
		(*b*) Describe any methods used to examine subgroups and interactions	6-7
		(*c*) Explain how missing data were addressed	6-7
		(*d*) If applicable, describe analytical methods taking account of sampling strategy	N/A
		(*e*) Describe any sensitivity analyses	6-7
**Results**			

For peer review only

Participants	13*	(a) Report numbers of individuals at each stage of study—eg numbers potentially eligible, examined for eligibility, confirmed eligible, included in the study, completing follow-up, and analysed	6, 8
		(b) Give reasons for non-participation at each stage	6, 8
		(c) Consider use of a flow diagram	N/A
Descriptive data	14*	(a) Give characteristics of study participants (eg demographic, clinical, social) and information on exposures and potential confounders	7, 9
		(b) Indicate number of participants with missing data for each variable of interest	6, 8
Outcome data	15*	Report numbers of outcome events or summary measures	7-10
Main results	16	(*a*) Give unadjusted estimates and, if applicable, confounder-adjusted estimates and their precision (eg, 95% confidence interval). Make clear which confounders were adjusted for and why they were included	7-10
		(*b*) Report category boundaries when continuous variables were categorized	7-10
		(*c*) If relevant, consider translating estimates of relative risk into absolute risk for a meaningful time period	N/A
Other analyses	17	Report other analyses done—eg analyses of subgroups and interactions, and sensitivity analyses	7-10
**Discussion**			
Key results	18	Summarise key results with reference to study objectives	11, 13
Limitations	19	Discuss limitations of the study, taking into account sources of potential bias or imprecision. Discuss both direction and magnitude of any potential bias	13
Interpretation	20	Give a cautious overall interpretation of results considering objectives, limitations, multiplicity of analyses, results from similar studies, and other relevant evidence	11-14
Generalisability	21	Discuss the generalisability (external validity) of the study results	11-14
**Other information**			
Funding	22	Give the source of funding and the role of the funders for the present study and, if applicable, for the original study on which the present article is based	15

*Give information separately for cases and controls in case-control studies and, if applicable, for exposed and unexposed groups in cohort and cross-sectional studies.

Note: An Explanation and Elaboration article discusses each checklist item and gives methodological background and published examples of transparent reporting. The STROBE checklist is best used in conjunction with this article (freely available on the Web sites of PLoS Medicine at http://www.plosmedicine.org/, Annals of Internal Medicine at http://www.annals.org/, and Epidemiology at http://www.epidem.com/). Information on the STROBE Initiative is available at www.strobe-statement.org.

For peer review only

BMJ Open

Investigation of time to first presentation and extra-hospital factors in the treatment of neovascular age-related macular degeneration (AMD): a retrospective cross-sectional study

Journal:	*BMJ Open*
Manuscript ID	bmjopen-2017-017771.R1
Article Type:	Research
Date Submitted by the Author:	10-Aug-2017
Complete List of Authors:	Sim, Peng Yong; The University of Edinburgh<br>Gajree, Sonul; Gartnavel General Hospital<br>Dhillon, Baljean; The University of Edinburgh; Princess Alexandra Eye Pavilion<br>Borooah, Shyamanga; Moorfields Eye Hospital
**Primary Subject Heading**:	Ophthalmology
Secondary Subject Heading:	Public health
Keywords:	OPHTHALMOLOGY, Medical retina < OPHTHALMOLOGY, PUBLIC HEALTH

SCHOLARONE™
Manuscripts

For peer review only

Investigation of time to first presentation and extra-hospital factors in the treatment of neovascular age-related macular degeneration (AMD): a retrospective cross-sectional study

Peng Yong Sim[1]
Sonul Gajree[2]
Baljean Dhillon[1,3]
Shyamanga Borooah[4]

[1]The University of Edinburgh, Edinburgh, UK
[2]Gartnavel General Hospital, Glasgow, UK
[3]The Princess Alexandra Eye Pavilion, Edinburgh, UK
[4]Moorfields Eye Hospital, London, UK

Corresponding author:
Name : Peng Yong Sim
Email : pengyong91@gmail.com

Conflict of interest: None

For peer review only

Abstract

Objectives: To assess the time from symptom onset to treatment for neovascular age-related macular degeneration (AMD) and to measure the awareness of AMD in south-east Scotland.

Design: Retrospective cross-sectional study.

Setting: Secondary care, south-east of Scotland.

Methods: Patients treated with intravitreal therapy (IVT) for neovascular AMD in south-east Scotland between 2013–2015 were identified using a treatment register. Notes were retrospectively reviewed. We measured time from A) symptom onset to first presentation at primary care, B) referral to ophthalmic clinic appointment and C) ophthalmic clinic appointment to first IVT treatment. To investigate AMD awareness, we performed a cluster random sample survey of patients visiting non-AMD ophthalmic clinics using a previously validated 12-item questionnaire.

Results: 195 patients (mean age 78) were included in the study. The mean delays between the different stages – A, B and C – were 54.2 (95% CI±13), 28.2 (95% CI±4.0) and 31.5 (95% CI±3.6) days respectively. There was an additional mean delay of 7.5 (95% CI±1.6) days when patients were indirectly referred by optometrists via general practitioners ($p<0.05$). 140 patients (mean age 78) participated in the awareness survey; 62.1% reported being “aware” of AMD but only 37.3% described AMD symptoms correctly.

For peer review only - http://bmjopen.bmj.com/site/about/guidelines.xhtml

Conclusions: There is significant delay at every step of the neovascular AMD patient pathway in south-east Scotland. Our findings suggest that suboptimal awareness of AMD symptoms could account for a substantial delay in presentation from symptom onset. This highlights the need to increase awareness of AMD which may optimise visual outcomes for patients.

Article Summary (Strengths and Limitations of this Study)

- Case notes of consecutive patients identified systematically using a treatment clinic register over 2 years.
- Demographic factors such as age, gender, education, social class and smoking status were taken into account for analyses of AMD awareness.
- Unable to ascertain direct association between low disease awareness and delay in treatment for patients with neovascular AMD as different cohort of patients were examined.
- Due to retrospective analysis of notes from a disease register in this study, the number of patients that could be included was limited by the quality of note keeping.

For peer review only

For peer review only - http://bmjopen.bmj.com/site/about/guidelines.xhtml

Introduction

Age-related macular degeneration (AMD) is the leading cause of vision loss in the developed world.[1] There are two main forms of AMD. The first is non-neovascular (dry) AMD which accounts for the majority of AMD cases and results from the deposition of drusen (small yellow or white deposits) underneath the retina that eventually leads to the slow degeneration of retinal cells resulting in blindness. Neovascular (wet) AMD accounts for the remaining cases of AMD and results from the development of new blood vessels deep to the retina which leak or bleed resulting in symptoms of new distortion or vision loss. Neovascular AMD results in irreversible blindness if left untreated and accounts for 90% of the cases of blind registration resulting from AMD.[2] The main risk factors associated with AMD are age and smoking.[3] Cases of blindness resulting from AMD are predicted to increase together with an ageing population.[4]

An effective treatment is currently available to preserve vision in neovascular AMD in the form of intravitreal therapy (IVT) with anti-vascular endothelial growth factor agents.[5–8] These drugs have been shown to be effective in maintaining long-term vision in the majority of patients affected by neovascular AMD.[9] Delay in instituting IVT treatment in new cases of neovascular AMD has been shown to be one of the most important factors negatively impacting final visual outcome.[10,11] Consequently, the early diagnosis and treatment is crucial to not only improving visual outcomes in AMD, but also to reduce the social and economic burden of blindness resulting from the disease.[12,13]

Delays from symptom onset to treatment can be experienced at different stages of the patient care pathway for new onset neovascular AMD. These include: 1) time of first symptom onset to presentation at primary care practitioner, 2) time from primary care referral to presentation at ophthalmic clinic and 3) time from ophthalmic clinic to first IVT treatment (Fig. 1). These early

For peer review only - http://bmjopen.bmj.com/site/about/guidelines.xhtml

stages of the care pathway also represent the periods during which lesions may be most active and amenable to the benefits of therapy.[14]

There have been many published reports investigating intra-hospital factors such as the time from first ophthalmic clinic visit to first IVT treatment.[10,11,16] However, there is a scarcity of literature reporting the extra-hospital factors such as the time from symptom onset to presentation at ophthalmic clinic. In addition, despite its significance in causing blindness, limited research has been performed to investigate AMD awareness. An exploration of patient's awareness and knowledge of disease has been demonstrated in other chronic diseases such as stroke and cancer[17,18], with increased awareness associated with improved patient outcomes.[19,20]

The primary objectives of this study were twofold; first, to assess the time between the different stages of the neovascular AMD care pathway in patients treated in south-east Scotland and second, to evaluate patients' awareness of AMD, its risk factors and treatment options.

Methods

Case notes of consecutive patients diagnosed and treated with IVT for neovascular AMD in NHS Lothian since September 2013 were identified using a treatment clinic register. A 2013 cut-off point was chosen to reflect the updated guidelines on AMD by the Royal College of Ophthalmologists (RCOphth) which were published at the time.[15] The guidelines recommended that all patients with suspected AMD should be seen by a retinal specialist within one week of referral, and that treatment should commence within one week of first ophthalmic appointment (Fig. 1).

For peer review only

For peer review only - http://bmjopen.bmj.com/site/about/guidelines.xhtml

In this study, the main outcome measures were 1) time from symptom onset to first presentation at primary care (i.e. duration of visual symptoms before initial presentation), 2) time from primary care referral to ophthalmic clinic appointment, and 3) time from ophthalmic clinic appointment to first IVT treatment. The main exclusion criteria were case notes with incomplete data and the co-existence of ocular comorbidities that gave rise to choroidal neovascularisation. This study was approved as part of a wider service evaluation which was accepted following review by the NHS Lothian Ophthalmology Quality Improvement Team on 8th October 2015.

In order to investigate patients' awareness of AMD, a cluster random sample of patients visiting ophthalmic clinics for non-AMD disease in NHS Lothian was surveyed using a 12-item questionnaire (see Supplementary File). The sample size required for the study was calculated using a power calculation (see Supplementary Data). Questions were adapted from a previously validated questionnaire[21] and served to ascertain each patient's knowledge of AMD and its risk factors. Patients were asked for their demographic details, including age, sex, education and postcode of residence. Socioeconomic deprivation scores (social class) were calculated for all patients from postcode data at the time of interview using the Scottish Index of Multiple Deprivation (SIMD).[22] The SIMD combines weighted data on seven domains (income, employment, education, housing, health, crime and geographical access) and is officially sanctioned by the Scottish Government as a measure of multiple deprivation.[23,24]

The first part of the questionnaire explored patients' familiarity with AMD and its risk factors. The second part enquired about patients' smoking status and their awareness of available treatments for AMD. Surveys were distributed and collected by the same researcher, who remained nearby to answer any questions about instructions. No additional assistance was provided. The survey was performed from 18th November 2015 to 31st November 2015 and data

For peer review only - http://bmjopen.bmj.com/site/about/guidelines.xhtml

was analysed using Pearson χ^2 tests except for education and social class where χ^2 tests for trend were performed. Data analysis was done using IBM SPSS Statistics for Windows, version 23 (IBM Corp., Armonk, N.Y., USA).

Results

Delay in presentation, referral and treatment of AMD in south-east Scotland

A total of 315 case notes were identified; 120 of the 315 were excluded after application of the exclusion criteria (see Supplementary Data for the demographics and breakdown of excluded cases), leaving 195 case notes for analysis. 120 (61.5%) patients were female, with a mean age of 78 years. Nearly all patients (187; 95.9%) presented with neovascular AMD affecting the first eye. The overall mean time from symptom onset to presentation was 54.2 (95% CI±13) days. As for referrals to ophthalmology, 118 (60.5%) of these were direct from optometrists, 5 (2.6%) were direct from GPs, and 52 (26.7%) were made by optometrists via GPs. The remaining referrals were from other hospitals, other ophthalmology clinics, and screening programmes.

The mean time from referral to ophthalmic clinic appointment was 28.2 days (95% CI±4.0 days). There was a significant additional mean delay of 7.5 ($p<0.05$) (95 %CI±1.6) days when patients were referred from their optometrist via their GP. During clinic appointments, fundus fluorescein angiogram was performed in approximately one third of patients (66/195). The mean time from clinic to first IVT treatment was 31.5 (95% CI±3.6) days (Fig. 2).

Awareness of AMD and its risk factors

The delay from symptom onset to first injection resulted from both intra-hospital and extra-hospital factors. We have already identified that when optometrists referred via the GPs instead of directly to the hospital eye service this resulted in a significant increased delay. However,

For peer review only - http://bmjopen.bmj.com/site/about/guidelines.xhtml

even this delay is overshadowed by the mean delay from symptom onset to presentation at primary care service. In order to better understand patient factors that may have resulted in this delay in presentation we performed a questionnaire survey on patients with unrelated disease in the eye service. A total of 142 patients were approached in non-AMD ophthalmic clinics. These clinics included glaucoma, ocular motility and general outpatient clinics. 140 patients agreed to participate. Two refused because of unwillingness and inability to understand the purpose of the questionnaire due to deafness respectively.

The cohort included 61 (43.6%) male and 79 (56.4%) female with a median age of 73 (range 17-93), comprising all social classes. The education level of patients ranged from primary education to university degree. Details of the demographic data are given in Table 1.

Table 1. Demographic data of patients (n=140)

Variable	*n*	%
Gender		
Male	61	43.6
Female	79	56.4
Age (years)		
<50	19	13.6
≥50	121	86.4
Highest education level attained		
Primary school	4	2.9%
Secondary school	78	55.7%
College	31	22.1%
University degree	27	19.3%
Social class		
I	13	9.3%
II	25	17.8%
III	20	14.3%
IV	27	19.3%
V	55	39.3%
Smoking status		
Current smoker	11	7.9%
Ex- or non-smoker	129	92.1%

Of the 140 respondents, 87 (62.1%) reported being "aware" of AMD. 14 (10%) had previously been diagnosed with AMD. 10 of these 14 patients (71.4%) were able to provide a correct

For peer review only - http://bmjopen.bmj.com/site/about/guidelines.xhtml

description of the symptoms of AMD. For those patients without a prior diagnosis, only 47/126 (37.3%) were able to correctly report the symptoms of AMD. There was a significant difference when comparing the responses of those who had a previous diagnosis of AMD to those without AMD (p=0.013). Overall female respondents were more likely than male respondents to report awareness of AMD (p=0.015) (Table 2). Increased awareness of AMD was also seen with higher levels of education (p=0.001).

Table 2. Respondents indicating awareness of AMD (n=140)

**Characteristic**[a]	**No. Indicating Awareness / Total No. (%)**
Gender distribution	
Male	31/61 (50.8)
Female	56/79 (70.9)
p value	0.015
Age (years)	
<50	8/19 (42.1)
≥50	79/121 (65.3)
p value	0.053
Highest education level attained	
Primary school	4/7 (57.1)
Secondary school	37/75 (49.3)
College	23/31 (74.2)
University degree	23/27 (85.2)
p value[b]	0.001
Social class	
I	8/13 (61.5)
II	18/25 (72.0)
III	9/20 (45.0)
IV	21/27 (77.8)
V	31/55 (56.4)
p value[b]	0.537
Smoking status	
Current smoker	6/11 (54.5)
Ex- or non-smoker	81/129 (62.8)
p value	0.588

[a] Unless otherwise indicated, *p* values are derived using the Pearson χ^2 test.
[b] Derived using the χ^2 for trend.

The top risk factor for AMD correctly considered by patients was age (127/140 - 90.7%). The other risk factors identified included smoking in 82 (58.6%), unprotected UV exposure in 62 (44.3%), genetic predisposition in 62 (44.3%), vitamin deficiency in 54 (38.6%) and gender in 15 (10.7%).

For peer review only - http://bmjopen.bmj.com/site/about/guidelines.xhtml

87 (62.1%) of patients thought that AMD was a treatable condition. However, only 20/87 (23%) were able to provide correct information on the available treatments (i.e. eye injections and laser therapy). The majority of patients (91/140, 65%) considered opticians to be their first port of call if they had vision problems. Other healthcare professionals cited as first port of call included general practitioners in 28 (20%) and ophthalmologists in 21 (15%).

Discussion

The Royal College of Ophthalmologists (RCOphth) has recently updated its guidance on suggested waiting times for IVT treatment in neovascular AMD in the hospital setting. It recommends that all patients should be seen by a retinal specialist within one week of primary care referral, and should begin treatment within one week following this.[15] The new guidelines place increased importance on correct diagnosis and urgent referral from primary care and place increasing emphasis on hospital eye services to provide capacity for urgent new AMD cases in addition to the treatment of existing neovascular AMD patients. However, this study finds that there are significant delays at each step of the neovascular AMD care pathway in south-east Scotland; both the waiting times from 1) primary care referral to ophthalmic clinic and 2) initial ophthalmic assessment to treatment are about four times as long as the recommended gold standard. In addition, there is a further one-week delay on average when indirect referrals are made by optometrists via GP. Similar findings have also been reported in previous studies which have demonstrated similar, if not longer, delays for intra-hospital pathways (i.e. from initial ophthalmic assessment to treatment).[10,11,16]

Delays from intra-hospital pathways may be attributed to the inherent diagnostic and referral pathways within different healthcare systems. In south-east Scotland, a new IT scheme linking community optometrists and eye clinics within hospitals across all of Scotland was introduced in

For peer review only - http://bmjopen.bmj.com/site/about/guidelines.xhtml

2010 following a successful pilot scheme in NHS Fife which allowed optometrists to make direct electronic referrals to ophthalmologists.[25] However the system has yet to be fully integrated into all units. Our study has highlighted that there is still much room for improvement for both the primary care referral system, and also within the acute referral clinics themselves. The current electronic system still relies on a manual, ad-hoc system for making referrals. An important step forward would be to develop a semi-automated referral system so that eye care providers can track patient referrals, obtain data on patient leakages and receive automatic notifications when there is lack of follow-up.

To our knowledge, this is the first study to evaluate the time from symptom onset to presentation at clinic (extra-hospital pathway) for patients with neovascular AMD in the UK. There are however several limitations to the study. First, assessment of presentation delay might be difficult due to the retrospective nature of evaluation of symptom onset by patients. Second, the perception of symptoms is also highly subjective, often depending on factors such as existing cognitive function, ocular dominance of the affected eye and baseline visual acuity of the unaffected eye. Nevertheless, it is noteworthy that this time interval often varies widely between patients and is prolonged in most cases. Therefore, although less accurate than formal angiographic diagnosis, we thought it is important to investigate this time interval as it would be accessible to intervention.

Our findings demonstrate that presentation delay not only represents a major source of delay but also accounts for the greatest proportion of the delay in the neovascular AMD care pathway in south-east Scotland. This represents an important target for improvement to reduce vision loss resulting from delay in the neovascular AMD care pathway.[26] This delay is likely to be complex and multifactorial, involving patients, eye care providers and healthcare systems. Barriers to early presentation might include a lack of awareness of AMD among patients, self-

For peer review only - http://bmjopen.bmj.com/site/about/guidelines.xhtml

examination by patients and screening of the disease by non-retina specialists. This can be further compounded by issues such as transport difficulties, age-related infirmity and a mismatch between patient expectations on speed of referral and recommended guidelines.[27]

At present, the diagnosis of new neovascular AMD, especially for the first affected eye, still very much relies on self-recognition of visual symptoms by patient themselves. This is however problematic as those affected in only one eye tend not to be aware of the visual change and may therefore remain “asymptomatic” for a considerable length of time.[28] Indeed, this seemed to be case in our study in which nearly all patients presented with neovascular AMD affecting the first eye.

There is evidence to show that the best corrected visual acuity at the time of diagnosis of neovascular AMD is worse for the first affected eye when compared to that of the second eye.[29] In addition, previous studies have shown that the visual prognosis of the first affected eye following one year of treatment is usually worse compared to that of the second affected eye in neovascular AMD.[30,31] These better outcomes of the second affected eye are most likely due to increased awareness and more frequent monitoring of the second eye as part of a systematic bilateral follow-up examination for the first affected eye. These factors would seemingly translate into a shorter delay in presentation for the second affected eye but it is should be noted that this association was not explored in our study and remains to be investigated. Nonetheless, the considerable delay in presentation for the first affected eye demonstrated in our study highlights the importance of early detection and treatment.

From the patient’s perspective, the delay in symptom recognition can be addressed to a certain extent by self-examination. Patients, especially those with an increased risk of developing neovascular AMD, should be educated and made aware of symptoms such as new visual

For peer review only

For peer review only - http://bmjopen.bmj.com/site/about/guidelines.xhtml

distortion and sudden reduction in vision. This can be achieved by encouraging patients to use suitable spaced self-tests of vision which examine one eye at a time to prevent compensation from the good eye. The standard Amsler test has long been recommended as the standard self-monitoring test but there has been increasing reservation about its utility as a diagnostic tool due to its insufficient reliability and variable sensitivity.[32,33] The advent of more innovative, cost saving technologies may circumvent these issues and make implementation of self-examination on a wider public scale more feasible in the near future.[34,35]

In this study, we chose to investigate patients' awareness of AMD because it is clear that a lack of disease awareness is a common factor for delayed presentation in other eye conditions such as glaucoma, retinal detachment and central retinal artery occlusion.[36,37] The only previous study to investigate AMD awareness in the UK population showed a low awareness (16%).[38] Our study adds to the existing literature by demonstrating that public awareness of AMD is still limited. Our survey shows that awareness of AMD is unacceptably low (37%), especially considering that this condition is the leading cause of blindness in developed countries.[1] The low awareness of AMD is also consistent with the low levels of awareness of AMD in other countries including Australia, Hong Kong, Singapore, Nepal, Bangladesh, China and the United States (range between 5% to 50.5%).[21,38,39–44] It is likely that our findings underestimate the true scale of lack of awareness among the general population because we sampled ophthalmic patients who, by virtue of being surveyed in an eye hospital, are presumably somewhat more attuned to common eye diseases. Our survey also highlights a low awareness of risk factors of AMD (other than age). However, this assessment could be limited by the lack of plausible distractors in the corresponding question which might have increased the respondent's chances of getting a correct answer(s), hence again underestimating the true scale of lack of awareness.

For peer review only - http://bmjopen.bmj.com/site/about/guidelines.xhtml

These findings are important given the severity of the consequences of delayed presentation in AMD and the ready availability of an effective treatment to prevent visual loss. We identified AMD-naive male patients and those with lower education levels to have a particularly low awareness of warning symptoms of AMD, suggesting the need for targeted intervention for these subgroups. As increased awareness can lead patients to seek appropriate medical care, improving awareness would logically lead to better visual prognoses for patients.

There is currently still a need for a unified national awareness campaign on AMD in the UK. A recent report by the Royal National Institute of Blind People highlighted that most initiatives at improving AMD awareness in the UK still operate at a local level.[27] Even then, these efforts often comprised of educational talks targeted at existing patients, rather than raising public awareness. The need for a national campaign has also been recognised by the Macular Society which has made increasing AMD awareness one of the main objectives of its five-year national strategy.[45]

Although some progress has been made since,[46] there is still room for improvement. Current awareness interventions need to be further optimised for a sustained impact. A promising step would be the adoption of the multi-layered approach as adopted by other developed countries.[47] This approach saw the use of a campaign which included a diverse range of activities such as promoting education programmes for patients and primary care, running a national advertising campaign and providing free mobile screening. The end of this focused campaign saw a dramatic increase in AMD awareness and the number of the population requesting fundus examination for symptoms of AMD.[47] The implementation of a similar public health strategy in the UK may achieve similar desirable effects but further research is needed to evaluate the effectiveness of this approach in the UK population. Another important gap highlighted by our study is the underappreciated link between smoking and AMD. This represents a potent novel

For peer review only - http://bmjopen.bmj.com/site/about/guidelines.xhtml

health promotional tool and awareness could be increased by incorporating information in existing campaigns with other smoking related diseases.[48,49]

Lack of awareness and knowledge of correct referral among non-ophthalmologists is also problematic. This may account for the delay in referral demonstrated in our study. A recent national survey revealed that 32% of GPs felt “de-skilled” in diagnosing common eye conditions.[50] The same survey also showed that 38% of GPs felt that eyes are the most difficult part of the body to diagnose. Achieving a better alignment of ophthalmic knowledge between healthcare organisations and professionals will help improve understanding and management of common ophthalmic disorders for those in the front line of eye care.

Conclusion

There is significant delay at every step of the care pathway for patients with neovascular AMD in south-east Scotland. We also show that awareness and knowledge of AMD are suboptimal. This lack of AMD could account for the long presentation delay of AMD to primary care. This suggests that efforts to educate the public regarding AMD may lead to earlier presentation and hence improved visual outcomes in patients.

Figure Legends

Fig 1. Flow chart depicting the typical care pathway of a patient with neovascular AMD in south-east Scotland. *Based on the recommendations by the Royal College of Ophthalmologists in its 2013 AMD guideline.[15]

Fig 2. Breakdown of the total delay (121.4 days) from symptom onset to treatment for patients with new neovascular AMD in south-east Scotland.

For peer review only - http://bmjopen.bmj.com/site/about/guidelines.xhtml

Acknowledgement

None

Funding

SB was sponsored by the Wellcome Trust Scottish Translational Medicine and Therapeutics Initiative (STMTI) scheme (grant number R42141) during the course of this research.

Competing interests

None declared.

Contributors

BD and SB were involved in conception and design of the study. PS and SG were involved in data acquisition, analysis and interpretation. PS was involved in first draft of manuscript. SG, SB and BD were involved in revising and critically appraising manuscript. PS, SG, BD and SB were involved in final approval for publication. BD and SB are guarantors.

References

1. World Health Organisation. Priority eye diseases. 2017. Available at: http://www.who.int/blindness/causes/priority/en/index7.html [Accessed January 19, 2017].

2. The Royal College of Ophthalmologists. Commissioning Contemporary AMD Services: A guide for commissioners and clinicians. 2007.

3. Smith W, Assink J, Klein R, et al. Risk factors for age-related macular degeneration: Pooled

For peer review only - http://bmjopen.bmj.com/site/about/guidelines.xhtml

findings from three continents. Ophthalmology. 2001; 108(4): 697–704.

4. Minassian DC, Reidy A, Lightstone A, et al. Modelling the prevalence of age-related macular degeneration (2010-2020) in the UK: expected impact of anti-vascular endothelial growth factor (VEGF) therapy. Br. J. Ophthalmol. 2011; 95(10): 1433–1436.

5. Brown DM, Kaiser PK, Michels M, et al. Ranibizumab versus verteporfin for neovascular age-related macular degeneration. N. Engl. J. Med. 2006; 355(14): 1432–44.

6. Brown DM, Michels M, Kaiser PK, et al. Ranibizumab versus Verteporfin Photodynamic Therapy for Neovascular Age-Related Macular Degeneration: Two-Year Results of the ANCHOR Study. Ophthalmology. 2009; 116(1): 57–65.e5.

7. Busbee BG, Ho AC, Brown DM, et al. Twelve-Month Efficacy and Safety of 0 . 5 mg or 2 . 0 mg Ranibizumab in Patients with Subfoveal Neovascular Age-related Macular. Ophthalmology. 2013: 1046–1056.

8. Heier JS, Brown DM, Chong V, et al. Intravitreal aflibercept (VEGF trap-eye) in wet age-related macular degeneration. Ophthalmology. 2012; 119(12): 2537–2548.

9. Rofagha S, Bhisitkul RB, Boyer DS, et al. Seven-year outcomes in ranibizumab-treated patients in ANCHOR, MARINA, and HORIZON: A multicenter cohort study (SEVEN-UP). Ophthalmology. 2013; 120(11): 2292–2299.

10. Lim JH, Wickremasinghe SS, Xie J, et al. Delay to treatment and visual outcomes in patients treated with anti-vascular endothelial growth factor for age-related macular degeneration. Am. J. Ophthalmol. 2012; 153(4): 678–686.e2.

11. Takahashi H, Ohkubo Y, Sato A, et al. Relationship Between Visual Prognosis and Delay of Intravitreal Injection of Ranibizumab When Treating Age-Related Macular Degeneration. Retina. 2015; 35(7): 1331–1338.

12. Bonastre J, Le Pen C, Soubrane G, et al. The burden of age-related macular degeneration: results of a cohort study in two French referral centres. Pharmacoeconomics. 2003; 21(3): 181–190.

For peer review only - http://bmjopen.bmj.com/site/about/guidelines.xhtml

13. Hopley C, Salkeld G, Wang JJ, et al. Cost utility of screening and treatment for early age related macular degeneration with zinc and antioxidants. Br. J. Ophthalmol. 2004; 88(4): 450–454.

14. Gonzales CR. Enhanced efficacy associated with early treatment of neovascular age-related macular degeneration with pegaptanib sodium: an exploratory analysis. Retina. 2005; 25(7): 815–27.

15. Chakravarthy U, Williams M, Amoaku W, et al. The Royal College of Ophthalmologists Guidelines on AMD: Executive Summary. Eye. 2013; 27(12): 1429–1431.

16. Arias L, Armadá F, Donate J, et al. Delay in treating age-related macular degeneration in Spain is associated with progressive vision loss. Eye. 2009; 23(2): 326–33.

17. Garraway WM, Whisnant JP. The changing pattern of hypertension and the declining incidence of stroke. JAMA. 1987; 258(2): 214–217.

18. Worden JK, Solomon LJ, Flynn BS, et al. A community-wide program in breast self-examination training and maintenance. Prev. Med. (Baltim). 1990; 19(3): 254–269.

19. Kim YS, Park SS, Bae HJ, et al. Stroke awareness decreases prehospital delay after acute ischemic stroke in Korea. BMC Neurol. 2011; 11: 2.

20. Niksic M, Rachet B, Duffy SW, et al. Is cancer survival associated with cancer symptom awareness and barriers to seeking medical help in England? An ecological study. Br. J. Cancer. 2016; 115(7): 876–886.

21. Livingston PM, McCarty CA, Taylor HR. Knowledge, attitudes, and self care practices associated with age related eye disease in Australia. Br. J. Ophthalmol. 1998; 82(7): 780–785.

22. The Scottish Government. Scottish Index of Multiple Deprivation 2012: A National Statistics Publication for Scotland; 2012. Available at: http://simd.scotland.gov.uk/publication-2012/

23. Aitken E, Dempster N, Ceresa C, et al. The impact of socioeconomic deprivation on outcomes following renal transplantation in the West of Scotland. Transplant. Proc. 2013; 45(6): 2176–2183.

For peer review only

24. Low L, Law JP, Hodson J, et al. Impact of socioeconomic deprivation on the development of diabetic retinopathy: a population-based, cross-sectional and longitudinal study over 12 years. BMJ Open. 2015; 5(4): e007290.

25. Cameron JR, Ahmed S, Curry P, et al. Impact of direct electronic optometric referral with ocular imaging to a hospital eye service. Eye (Lond). 2009; 23(5): 1134–40.

26. Rasmussen A, Brandi S, Fuchs J, et al. Visual outcomes in relation to time to treatment in neovascular age-related macular degeneration. Acta Ophthalmol. 2015; 93(7): 616–620.

27. Royal National Institute of Blind People. Don't lose sight! Don't delay! Perspectives on the wet age-related macular degeneration (wet AMD) patient journey. RNIB Campaign Report. 2013. Available at: https://www.rnib.org.uk/sites/default/files/Don%27t%20lose%20sight%20don%27t%20delay%20Campaign%20report.pdf [Accessed April 15, 2017]

28. Bressler NM. Early detection and treatment of neovascular age-related macular degeneration. J. Am. Board Fam. Pract. 2002; 15(2): 142–52.

29. Chevreaud O, Semoun O, Blanco-Garavito R, et al. Visual acuity at presentation in the second eye versus first eye in patients with exudative age-related macular degeneration. Eur. J. Ophthalmol. 2015; 26(1): 44–47.

30. Muniraju R, Jayashree R, Sivaprasad S. Outcomes of Second eye Versus First eye in Patients Treated with Ranibizumab for Bilateral Neovascular Age Related Macular Degeneration. Invest. Ophthalmol. Vis. Sci. 2013; 54(15): 2013.

31. Chew J, Broadhead G, Li H, et al. Visual outcomes of ranibizumab treatment in fellow eyes of exudative AMD. Invest. Ophthalmol. Vis. Sci. 2013; 54(15): 3832.

32. Zaidi FH, Cheong-Leen R, Gair EJ, et al. The Amsler chart is of doubtful value in retinal screening for early laser therapy of subretinal membranes. The West London Survey. Eye (Lond). 2004; 18(5): 503–8.

33. Faes L, Bodmer NS, Bachmann LM, et al. Diagnostic accuracy of the Amsler grid and the preferential hyperacuity perimetry in the screening of patients with age-related macular

For peer review only

For peer review only - http://bmjopen.bmj.com/site/about/guidelines.xhtml

degeneration: systematic review and meta-analysis. Eye (Lond). 2014; 28(7): 788–96.

34. Winther C, Frisén L. Self-Testing of Vision in Age-Related Macula Degeneration: A Longitudinal Pilot Study Using a Smartphone-Based Rarebit Test, Self-Testing of Vision in Age-Related Macula Degeneration: A Longitudinal Pilot Study Using a Smartphone-Based Rarebit Test. J. Ophthalmol. J. Ophthalmol. 2015; 2015, 2015: e285463.

35. Schwartz R, Loewenstein A. Early detection of age related macular degeneration: current status. Int. J. Retin. Vitr. 2015; 1(1): 1–8.

36. Saw S-M, Gazzard G, Friedman D, et al. Awareness of glaucoma, and health beliefs of patients suffering primary acute angle closure. Br. J. Ophthalmol. 2003; 87(4): 446–449.

37. Uhr JH, Mishra K, Wei C, et al. Awareness and knowledge of emergent ophthalmic disease among patients in an internal medicine clinic. JAMA Ophthalmol. 2016; 134(4): 424–431.

38. AMD Alliance International. Awareness of age-related macular degeneration and associated risk factors. AMD Global Report. 2005; Available at: http://www.eyeresearch.org/pdf/AMD_Report_Aug22_1046.pdf

39. Lau J, Lee V, Fan D, et al. Knowledge about cataract, glaucoma, and age related macular degeneration in the Hong Kong Chinese population. Br. J. Ophthalmol. 2002; 86: 1080–1084.

40. Sanjay S, Neo HY, Sangtam T, et al. Survey on the knowledge of age-related macular degeneration and its risk factors among Singapore residents. Clin. Exp. Ophthalmol. 2009; 37(8): 795–800.

41. Thapa R, Bajimaya S, Paudyal G, et al. Population awareness of diabetic eye disease and age related macular degeneration in Nepal: the Bhaktapur Retina Study. BMC Ophthalmol. 2015; 15(1): 188.

42. Islam FMA, Chakrabarti R, Islam SZ, et al. Factors associated with awareness, attitudes and practices regarding common eye diseases in the general population in a rural district in Bangladesh: The Bangladesh population-based diabetes and eye study (BPDES). PLoS One. 2015; 10(7): 1–12.

For peer review only - http://bmjopen.bmj.com/site/about/guidelines.xhtml

43. Scott AW, Bressler NM, Ffolkes S, et al. Public Attitudes About Eye and Vision Health. JAMA Ophthalmol. 2016; 21287(10): 1–8.

44. Zhang C, Zhang G, Ma N, et al. Awareness of Age-related Macular Degeneration and Its Risk Factors among Beijing Residents in China. 2017; 130(2): 155–159.

45. Macular Society. Strategy 2016-2020. 2016. Available at: https://www.macularsociety.org/sites/default/files/Macular%20Society%20strategy%202016-2020.pdf [Accessed April 15, 2017]

46. Royal National Insitute of Blind People. Charity announces new awareness week for macular conditions. 2015. Available at: http://www.rnib.org.uk/charity-announces-new-awareness-week-macular-conditions [Accessed April 15, 2017]

47. Heraghty J, Cummins R. A layered approach to raising public awareness of Macular Degeneration in Australia. Am. J. Public Health. 2012; 102(9): 1655–1659.

48. Thornton J, Edwards R, Harrison RA, et al. 'Smoke gets in your eyes': A research-informed professional education and advocacy programme. J. Public Health (Bangkok). 2007; 29(2): 142–146.

49. Thompson C, Harrison RA, Wilkinson SC, et al. Attitudes of community optometrists to smoking cessation: An untapped opportunity overlooked? Ophthalmic Physiol. Opt. 2007; 27(4): 389–393.

50. Optegra Eye Health Care Vision of Britain Report. An insight into the nation's eye health. Optegra Eye Health Care. 2015. Available at: https://www.optegra.com/vob-report.pdf

For peer review only

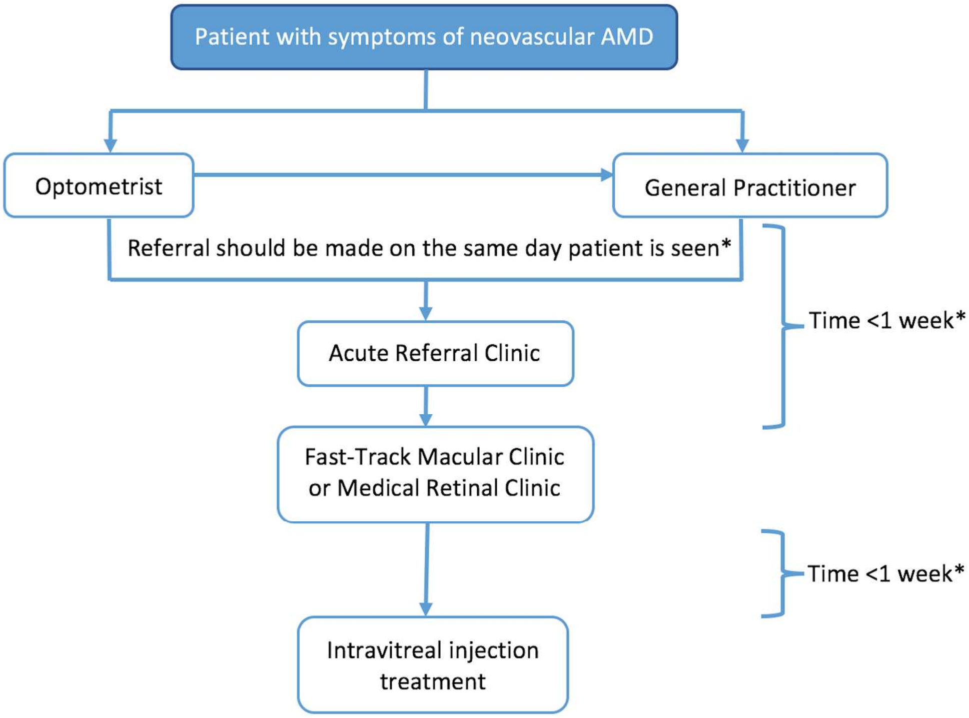

Fig 1. Flow chart depicting the typical care pathway of a patient with neovascular AMD in south-east Scotland. *Based on the recommendations by the Royal College of Ophthalmologists in its 2013 AMD guideline.[15]

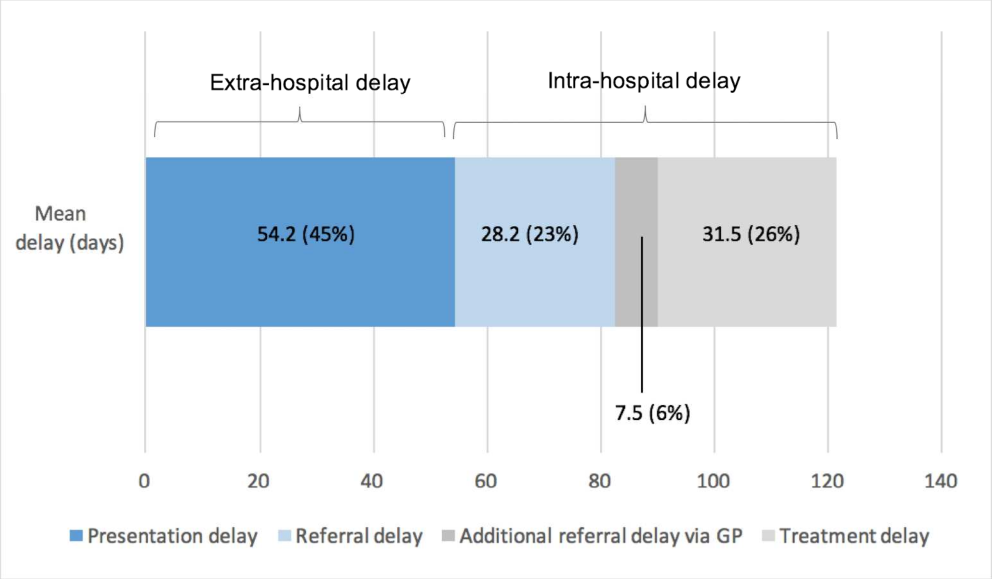

Fig 2. Breakdown of the total delay (121.4 days) from symptom onset to treatment for patients with new neovascular AMD in south-east Scotland.

review only

Appendix 1: AMD Awareness Questionnaire.

No: Postcode:

AMD Awareness Questionnaire

Please circle the correct option

1. **Sex:** Male Female

2. **Age (years):**

3. **Educational Status:**
   1) Completed Primary School
   2) Completed Secondary School
   3) College Qualification
   4) University Degree
   5) Prefer not to say

4. **Employment status:**
   1) Unemployed
   2) Full time
   3) Part time
   4) Retired
   5) Other (specify):
   6) Prefer not to say

5. **Prior to now, have you ever been told you have Age Related Macular Degeneration (AMD)?**
   1) Yes
   2) No

IF 'Yes', MOVE ON TO QUESTION 7

6. **If no, have you ever heard of Age Related Macular Degeneration (AMD)?**
   1) Yes
   2) N

For peer review only

7. **If you have heard of AMD, can you describe the condition and its symptoms?**

____

____

8. **Regardless of whether or not you are familiar with AMD, which of the following factors do you think increases the risk of developing AMD? (select all that apply)**

   1) Smoking
   2) Vitamin deficiency
   3) Age
   4) Unprotected exposure to sunlight
   5) Genetics
   6) Sex

9. **Do you currently smoke?**

   1) Yes
   2) No

10. **Is AMD a treatable condition?**

   1) Yes
   2) No

11. **If yes, do you know what treatments are available?**

____

____

12. **If you were worried about your eyesight, where would you go for advice**?

   1) Ophthalmologist
   2) GP
   3) Pharmacist
   4) Optician
   5) Other (specify):

For peer review only

Appendix 2: Power calculation for AMD awareness survey sample size.

**Sample Size:X-Sectional, Cohort, & Randomized Clinical Trials**			
Two-sided significance level(1-alpha):			95
Power(1-beta, % chance of detecting):			80
Ratio of sample size, Unexposed/Exposed:			1
Percent of Exposed with Outcome:			25
Odds Ratio:			6.3
Risk/Prevalence Ratio:			5
Risk/Prevalence difference:			20
	**Kelsey**	**Fleiss**	**Fleiss with CC**
Sample Size - Exposed	51	49	59
Sample Size-Nonexposed	51	49	59
Total sample size:	102	98	118

References

Kelsey et al., Methods in Observational Epidemiology 2nd Edition, Table 12-15
Fleiss, Statistical Methods for Rates and Proportions, formulas 3.18 &3.19
CC = continuity correction

Based on a global survey investigating AMD awareness by AMD Alliance International, the level of awareness in the UK was 16% in 2005.[38] Allowing for increase in awareness over time (demonstrated by studies in other countries), hence assuming a slightly higher level of awareness (~25%) in south-east Scotland, we would have a power of 80% to detect this with a total sample size of 118 patients.

Appendix 3: Table comparing summary demographic data for included vs excluded case notes.

**Patient demographics**	**Analysed (*n* = 195)**	**Excluded (*n* = 120)**
Sex (% female)	61.5	58.3
Mean age (years)	77.7	78.4
Percentage of patients presenting with first affected eye	95.9%	97.5%

Appendix 4: Table showing breakdown of case notes excluded from study.

**Total case notes identified**	**315**
Co-existence of ocular comorbidities that give rise to choroidal neovascularization	23
Symptom duration not recorded	76
Lost to follow-up	21
**Case notes included in study**	**195**

STROBE 2007 (v4) Statement—Checklist of items that should be included in reports of *cross-sectional studies*

Section/Topic	Item #	Recommendation	Reported on page #
**Title and abstract**	1	(*a*) Indicate the study's design with a commonly used term in the title or the abstract	1
		(*b*) Provide in the abstract an informative and balanced summary of what was done and what was found	2
**Introduction**			
Background/rationale	2	Explain the scientific background and rationale for the investigation being reported	4-5
Objectives	3	State specific objectives, including any prespecified hypotheses	6
**Methods**			
Study design	4	Present key elements of study design early in the paper	6
Setting	5	Describe the setting, locations, and relevant dates, including periods of recruitment, exposure, follow-up, and data collection	6
Participants	6	(*a*) Give the eligibility criteria, and the sources and methods of selection of participants	6
Variables	7	Clearly define all outcomes, exposures, predictors, potential confounders, and effect modifiers. Give diagnostic criteria, if applicable	6
Data sources/ measurement	8*	For each variable of interest, give sources of data and details of methods of assessment (measurement). Describe comparability of assessment methods if there is more than one group	6
Bias	9	Describe any efforts to address potential sources of bias	6-7
Study size	10	Explain how the study size was arrived at	6-7
Quantitative variables	11	Explain how quantitative variables were handled in the analyses. If applicable, describe which groupings were chosen and why	6-7
Statistical methods	12	(*a*) Describe all statistical methods, including those used to control for confounding	6-7
		(*b*) Describe any methods used to examine subgroups and interactions	6-7
		(*c*) Explain how missing data were addressed	6-7
		(*d*) If applicable, describe analytical methods taking account of sampling strategy	N/A
		(*e*) Describe any sensitivity analyses	6-7
**Results**			

For peer review only

Participants	13*	(a) Report numbers of individuals at each stage of study—eg numbers potentially eligible, examined for eligibility, confirmed eligible, included in the study, completing follow-up, and analysed	6, 8
		(b) Give reasons for non-participation at each stage	6, 8
		(c) Consider use of a flow diagram	N/A
Descriptive data	14*	(a) Give characteristics of study participants (eg demographic, clinical, social) and information on exposures and potential confounders	7, 9
		(b) Indicate number of participants with missing data for each variable of interest	6, 8
Outcome data	15*	Report numbers of outcome events or summary measures	7-10
Main results	16	(*a*) Give unadjusted estimates and, if applicable, confounder-adjusted estimates and their precision (eg, 95% confidence interval). Make clear which confounders were adjusted for and why they were included	7-10
		(*b*) Report category boundaries when continuous variables were categorized	7-10
		(*c*) If relevant, consider translating estimates of relative risk into absolute risk for a meaningful time period	N/A
Other analyses	17	Report other analyses done—eg analyses of subgroups and interactions, and sensitivity analyses	7-10
**Discussion**			
Key results	18	Summarise key results with reference to study objectives	11, 13
Limitations	19	Discuss limitations of the study, taking into account sources of potential bias or imprecision. Discuss both direction and magnitude of any potential bias	13
Interpretation	20	Give a cautious overall interpretation of results considering objectives, limitations, multiplicity of analyses, results from similar studies, and other relevant evidence	11-14
Generalisability	21	Discuss the generalisability (external validity) of the study results	11-14
**Other information**			
Funding	22	Give the source of funding and the role of the funders for the present study and, if applicable, for the original study on which the present article is based	15

*Give information separately for cases and controls in case-control studies and, if applicable, for exposed and unexposed groups in cohort and cross-sectional studies.

Note: An Explanation and Elaboration article discusses each checklist item and gives methodological background and published examples of transparent reporting. The STROBE checklist is best used in conjunction with this article (freely available on the Web sites of PLoS Medicine at http://www.plosmedicine.org/, Annals of Internal Medicine at http://www.annals.org/, and Epidemiology at http://www.epidem.com/). Information on the STROBE Initiative is available at www.strobe-statement.org.

For peer review only

Investigation of time to first presentation and extra-hospital factors in the treatment of neovascular age-related macular degeneration: a retrospective cross-sectional study

Journal:	*BMJ Open*
Manuscript ID	bmjopen-2017-017771.R2
Article Type:	Research
Date Submitted by the Author:	25-Sep-2017
Complete List of Authors:	Sim, Peng Yong; The University of Edinburgh<br>Gajree, Sonul; Gartnavel General Hospital<br>Dhillon, Baljean; The University of Edinburgh; Princess Alexandra Eye Pavilion<br>Borooah, Shyamanga; Moorfields Eye Hospital
**Primary Subject Heading**:	Ophthalmology
Secondary Subject Heading:	Public health
Keywords:	Medical retina < OPHTHALMOLOGY, PUBLIC HEALTH, age-related macular degeneration, neovascular AMD, AMD awareness

SCHOLARONE™
Manuscripts

Investigation of time to first presentation and extra-hospital factors in the treatment of neovascular age-related macular degeneration: a retrospective cross-sectional study

Peng Yong Sim[1]
Sonul Gajree[2]
Baljean Dhillon[1,3]
Shyamanga Borooah[4]

[1]The University of Edinburgh, Edinburgh, UK
[2]Gartnavel General Hospital, Glasgow, UK
[3]The Princess Alexandra Eye Pavilion, Edinburgh, UK
[4]Moorfields Eye Hospital, London, UK

Corresponding author:
Name : Peng Yong Sim
Email : pengyong91@gmail.com

Conflict of interest: None

For peer review only

Abstract

Objectives: To assess the time from symptom onset to treatment for neovascular age-related macular degeneration (AMD) and to measure the awareness of AMD in south-east Scotland.

Design: Retrospective cross-sectional study.

Setting: Secondary care, south-east of Scotland.

Methods: Patients treated with intravitreal therapy (IVT) for neovascular AMD (nvAMD) in south-east Scotland between 2013–2015 were identified using a treatment register. Notes were retrospectively reviewed. We measured time from A) symptom onset to first presentation at primary care, B) referral to ophthalmic clinic appointment and C) ophthalmic clinic appointment to first IVT treatment. To investigate AMD awareness, we performed a cluster random sample survey of patients visiting non-AMD ophthalmic clinics using a previously validated 12-item questionnaire.

Results: 195 patients (mean age 78) were included in the study. The mean delays between the different stages – A, B and C – were 54.2 (95% CI±13), 28.2 (95% CI±4.0) and 31.5 (95% CI±3.6) days respectively. There was an additional mean delay of 7.5 (95% CI±1.6) days when patients were indirectly referred by optometrists via general practitioners ($p<0.05$). 140 patients (mean age 78) participated in the awareness survey; 62.1% reported being “aware” of AMD but only 37.3% described AMD symptoms correctly.

For peer review only - http://bmjopen.bmj.com/site/about/guidelines.xhtml

Conclusions: There was a significant delay at every step of the nvAMD patient pathway. The causes for this were multifactorial and included delays in first presentation to a healthcare provider, referral from primary care and initiation of secondary care treatment. Our data is likely to underestimate pre-hospital delays as a large number of cases are likely to have undefined symptoms and onset. We also identified suboptimal awareness of AMD which could account for a substantial delay in presentation from symptom onset. These findings highlight the need to address AMD awareness and the need for urgent treatment to prevent avoidable vision loss resulting from nvAMD.

Article Summary (Strengths and Limitations of this Study)

- Case notes of consecutive patients identified systematically using a treatment clinic register over 2 years.
- Demographic factors such as age, gender, education, social class and smoking status were taken into account for analyses of AMD awareness.
- Unable to ascertain direct association between low disease awareness and delay in treatment for patients with nvAMD as different cohort of patients were examined.
- Due to retrospective analysis of notes from a disease register in this study, the number of patients that could be included was limited by the quality of note keeping.

For peer review only

Introduction

Age-related macular degeneration (AMD) is the leading cause of vision loss in the developed world.[1] There are two main forms of AMD. The first is non-neovascular (dry) AMD which accounts for the majority of AMD cases and results from the deposition of drusen (small yellow or white deposits) underneath the retina that eventually leads to the slow degeneration of retinal cells resulting in blindness. Neovascular (wet) AMD (nvAMD) accounts for the remaining cases of AMD and results from the development of new blood vessels deep to the retina which leak or bleed resulting in symptoms of new distortion or vision loss. nvAMD results in irreversible blindness if left untreated and accounts for 90% of the cases of blind registration resulting from AMD.[2] The main risk factors associated with AMD are age and smoking.[3] Cases of blindness resulting from AMD are predicted to increase together with an ageing population.[4]

An effective treatment is currently available to preserve vision in nvAMD in the form of intravitreal therapy (IVT) with anti-vascular endothelial growth factor agents.[5–8] These drugs have been shown to be effective in maintaining long-term vision in the majority of patients affected by nvAMD.[9] Delay in instituting IVT treatment in new cases of nvAMD has been shown to be one of the most important factors negatively impacting final visual outcome.[10,11] Consequently, the early diagnosis and treatment is crucial to not only improving visual outcomes in AMD, but also to reduce the social and economic burden of blindness resulting from the disease.[12,13]

Delays from symptom onset to treatment can be experienced at different stages of the patient care pathway for new onset nvAMD. These include: 1) time of first symptom onset to presentation at primary care practitioner, 2) time from primary care referral to presentation at ophthalmic clinic and 3) time from ophthalmic clinic to first IVT treatment (Fig. 1).[14] These early

For peer review only - http://bmjopen.bmj.com/site/about/guidelines.xhtml

stages of the care pathway also represent the periods during which lesions may be most active and amenable to the benefits of therapy.[15]

There have been many published reports investigating intra-hospital factors such as the time from first ophthalmic clinic visit to first IVT treatment.[10,11,16] However, there is a scarcity of literature reporting the extra-hospital factors such as the time from symptom onset to presentation at ophthalmic clinic. In addition, despite its significance in causing blindness, limited research has been performed to investigate AMD awareness. An exploration of patient's awareness and knowledge of disease has been demonstrated in other chronic diseases such as stroke and cancer[17,18], with increased awareness associated with improved patient outcomes.[19,20]

The primary objectives of this study were twofold; first, to assess the time between the different stages of the nvAMD care pathway in patients treated in south-east Scotland and second, to evaluate patients' awareness of AMD, its risk factors and treatment options.

Methods

Case notes of consecutive patients diagnosed and treated with IVT for nvAMD in NHS Lothian since September 2013 were identified using a treatment clinic register. A 2013 cut-off point was chosen to reflect the updated guidelines on AMD by the Royal College of Ophthalmologists (RCOphth) which were published at the time.[14] The guidelines recommended that all patients with suspected AMD should be seen by a retinal specialist within one week of referral, and that treatment should commence within one week of first ophthalmic appointment (Fig. 1).

In this study, the main outcome measures were 1) time from symptom onset to first presentation at primary care (i.e. duration of visual symptoms before initial presentation), 2) time from

For peer review only - http://bmjopen.bmj.com/site/about/guidelines.xhtml

primary care referral to ophthalmic clinic appointment, and 3) time from ophthalmic clinic appointment to first IVT treatment. The main exclusion criteria were case notes with incomplete data and the co-existence of ocular comorbidities that gave rise to choroidal neovascularisation. This study was approved as part of a wider service evaluation which was accepted following review by the NHS Lothian Ophthalmology Quality Improvement Team on 8th October 2015.

In order to investigate patients' awareness of AMD, a cluster random sample of patients visiting ophthalmic clinics for non-AMD disease in NHS Lothian was surveyed using a 12-item questionnaire (see Supplementary File). The sample size required for the study was calculated using a power calculation (see Supplementary Data). Questions were adapted from a previously validated questionnaire[21] and served to ascertain each patient's knowledge of AMD and its risk factors. Patients were asked for their demographic details, including age, sex, education and postcode of residence. Socioeconomic deprivation scores (social class) were calculated for all patients from postcode data at the time of interview using the Scottish Index of Multiple Deprivation (SIMD).[22] The SIMD combines weighted data on seven domains (income, employment, education, housing, health, crime and geographical access) and is officially sanctioned by the Scottish Government as a measure of multiple deprivation.[23,24]

The first part of the questionnaire explored patients' familiarity with AMD and its risk factors. The second part enquired about patients' smoking status and their awareness of available treatments for AMD. Surveys were distributed and collected by the same researcher, who remained nearby to answer any questions about instructions. No additional assistance was provided. The survey was performed from 18th November 2015 to 31st November 2015 and data was analysed using Pearson χ^2 tests except for education and social class where χ^2 tests for

For peer review only - http://bmjopen.bmj.com/site/about/guidelines.xhtml

trend were performed. Data analysis was done using IBM SPSS Statistics for Windows, version 23 (IBM Corp., Armonk, N.Y., USA).

Results

Delay in presentation, referral and treatment of AMD in south-east Scotland

A total of 315 case notes were identified; 120 of the 315 were excluded after application of the exclusion criteria (see Supplementary Data for the demographics and breakdown of excluded cases), leaving 195 case notes for analysis. 120 (61.5%) patients were female, with a mean age of 78 years. Nearly all patients (187; 95.9%) presented with nvAMD affecting the first eye. The overall mean time from symptom onset to presentation was 54.2 (95% CI±13) days. As for referrals to ophthalmology, 118 (60.5%) of these were direct from optometrists, 5 (2.6%) were direct from GPs, and 52 (26.7%) were made by optometrists via GPs. The remaining referrals were from other hospitals, other ophthalmology clinics, and screening programmes.

The mean time from referral to ophthalmic clinic appointment was 28.2 days (95% CI±4.0 days). There was a significant additional mean delay of 7.5 ($p<0.05$) (95 %CI±1.6) days when patients were referred from their optometrist via their GP. During clinic appointments, fundus fluorescein angiogram was performed in approximately one third of patients (66/195). The mean time from clinic to first IVT treatment was 31.5 (95% CI±3.6) days (Fig. 2).

Awareness of AMD and its risk factors

The delay from symptom onset to first injection resulted from both intra-hospital and extra-hospital factors. We have already identified that when optometrists referred via the GPs instead of directly to the hospital eye service this resulted in a significant increased delay. However, even this delay is overshadowed by the mean delay from symptom onset to presentation at

For peer review only - http://bmjopen.bmj.com/site/about/guidelines.xhtml

primary care service. In order to better understand patient factors that may have resulted in this delay in presentation we performed a questionnaire survey on patients with unrelated disease in the eye service. A total of 142 patients were approached in non-AMD ophthalmic clinics. These clinics included glaucoma, ocular motility and general outpatient clinics. 140 patients agreed to participate. Two refused because of unwillingness and inability to understand the purpose of the questionnaire due to deafness respectively.

The cohort included 61 (43.6%) male and 79 (56.4%) female with a median age of 73 (range 17-93), comprising all social classes. The education level of patients ranged from primary education to university degree. Details of the demographic data are given in Table 1.

Table 1. Demographic data of patients (n=140)

**Variable**	***n***	**%**
Gender		
Male	61	43.6
Female	79	56.4
Age (years)		
<50	19	13.6
≥50	121	86.4
Highest education level attained		
Primary school	4	2.9%
Secondary school	78	55.7%
College	31	22.1%
University degree	27	19.3%
Social class		
I	13	9.3%
II	25	17.8%
III	20	14.3%
IV	27	19.3%
V	55	39.3%
Smoking status		
Current smoker	11	7.9%
Ex- or non-smoker	129	92.1%

Of the 140 respondents, 87 (62.1%) reported being “aware” of AMD. 14 (10%) had previously been diagnosed with AMD. 10 of these 14 patients (71.4%) were able to provide a correct description of the symptoms of AMD. For those patients without a prior diagnosis, only 47/126

For peer review only - http://bmjopen.bmj.com/site/about/guidelines.xhtml

(37.3%) were able to correctly report the symptoms of AMD. There was a significant difference when comparing the responses of those who had a previous diagnosis of AMD to those without AMD (p=0.013). Overall female respondents were more likely than male respondents to report awareness of AMD (p=0.015) (Table 2). Increased awareness of AMD was also seen with higher levels of education (p=0.001).

Table 2. Respondents indicating awareness of AMD (n=140)

**Characteristic**[a]	**No. Indicating Awareness / Total No. (%)**
Gender distribution	
Male	31/61 (50.8)
Female	56/79 (70.9)
p value	0.015
Age (years)	
<50	8/19 (42.1)
≥50	79/121 (65.3)
p value	0.053
Highest education level attained	
Primary school	4/7 (57.1)
Secondary school	37/75 (49.3)
College	23/31 (74.2)
University degree	23/27 (85.2)
p value[b]	0.001
Social class	
I	8/13 (61.5)
II	18/25 (72.0)
III	9/20 (45.0)
IV	21/27 (77.8)
V	31/55 (56.4)
p value[b]	0.537
Smoking status	
Current smoker	6/11 (54.5)
Ex- or non-smoker	81/129 (62.8)
p value	0.588

[a] Unless otherwise indicated, *p* values are derived using the Pearson χ^2 test.
[b] Derived using the χ^2 for trend.

The top risk factor for AMD correctly considered by patients was age (127/140 - 90.7%). The other risk factors identified included smoking in 82 (58.6%), unprotected UV exposure in 62 (44.3%), genetic predisposition in 62 (44.3%), vitamin deficiency in 54 (38.6%) and gender in 15 (10.7%).

87 (62.1%) of patients thought that AMD was a treatable condition. However, only 20/87 (23%)

For peer review only - http://bmjopen.bmj.com/site/about/guidelines.xhtml

were able to provide correct information on the available treatments (i.e. eye injections and laser therapy). The majority of patients (91/140, 65%) considered opticians to be their first port of call if they had vision problems. Other healthcare professionals cited as first port of call included general practitioners in 28 (20%) and ophthalmologists in 21 (15%).

Discussion

The RCOphth has recently updated its guidance on suggested waiting times for IVT treatment in nvAMD in the hospital setting. It recommends that all patients should be seen by a retinal specialist within one week of primary care referral, and should begin treatment within one week following this.[14] The new guidelines place increased importance on correct diagnosis and urgent referral from primary care and place increasing emphasis on hospital eye services to provide capacity for urgent new AMD cases in addition to the treatment of existing nvAMD patients. However, this study finds that there are significant delays at each step of the nvAMD care pathway in south-east Scotland; both the waiting times from 1) primary care referral to ophthalmic clinic and 2) initial ophthalmic assessment to treatment are about four times as long as the recommended gold standard. In addition, there is a further one-week delay on average when indirect referrals are made by optometrists via GP. Similar findings have also been reported in previous studies which have demonstrated similar, if not longer, delays for intra-hospital pathways (i.e. from initial ophthalmic assessment to treatment).[10,11,16]

Delays from intra-hospital pathways may be attributed to the inherent diagnostic and referral pathways within different healthcare systems. In south-east Scotland, a new IT scheme linking community optometrists and eye clinics within hospitals across all of Scotland was introduced in 2010 following a successful pilot scheme in NHS Fife which allowed optometrists to make direct electronic referrals to ophthalmologists.[25] However the system has yet to be fully integrated into

For peer review only - http://bmjopen.bmj.com/site/about/guidelines.xhtml

all units. Our study has highlighted that there is still much room for improvement for both the primary care referral system, and also within the acute referral clinics themselves. The current electronic system still relies on a manual, ad-hoc system for making referrals. An important step forward would be to develop a semi-automated referral system so that eye care providers can track patient referrals, obtain data on patient leakages and receive automatic notifications when there is lack of follow-up.

To our knowledge, this is the first study to evaluate the time from symptom onset to presentation at clinic (extra-hospital pathway) for patients with nvAMD in the UK. There are however several limitations to the study. First, assessment of presentation delay might be difficult due to the retrospective nature of evaluation of symptom onset by patients. Second, the perception of symptoms is also highly subjective, often depending on factors such as existing cognitive function, ocular dominance of the affected eye and baseline visual acuity of the unaffected eye. Nevertheless, it is noteworthy that this time interval often varies widely between patients and is prolonged in most cases. Therefore, although less accurate than formal angiographic diagnosis, we thought it is important to investigate this time interval as it would be accessible to intervention.

Our findings demonstrate that presentation delay not only represents a major source of delay but also accounts for the greatest proportion of the delay in the nvAMD care pathway in south-east Scotland. This represents an important target for improvement to reduce vision loss resulting from delay in the nvAMD care pathway.[26] This delay is likely to be complex and multifactorial, involving patients, eye care providers and healthcare systems. Barriers to early presentation might include a lack of awareness of AMD among patients, self-examination by patients and screening of the disease by non-retina specialists. This can be further compounded by issues such as transport difficulties, age-related infirmity and a mismatch between patient

For peer review only - http://bmjopen.bmj.com/site/about/guidelines.xhtml
For peer review only

expectations on speed of referral and recommended guidelines.[27] Further studies are warranted into the reasons underlying our findings in both primary care and hospital eye service environments in order that appropriate measures are taken to identify patients early and build service capacity accordingly.

At present, the diagnosis of new nvAMD, especially for the first affected eye, still very much relies on self-recognition of visual symptoms by patient themselves. This is however problematic as those affected in only one eye tend not to be aware of the visual change and may therefore remain "asymptomatic" for a considerable length of time.[28] Indeed, this seemed to be case in our study in which nearly all patients presented with nvAMD affecting the first eye.

There is evidence to show that the best corrected visual acuity at the time of diagnosis of nvAMD is worse for the first affected eye when compared to that of the second eye.[29] In addition, previous studies have shown that the visual prognosis of the first affected eye following one year of treatment is usually worse compared to that of the second affected eye in nvAMD.[30,31] These better outcomes of the second affected eye are most likely due to increased awareness and more frequent monitoring of the second eye as part of a systematic bilateral follow-up examination for the first affected eye. These factors would seemingly translate into a shorter delay in presentation for the second affected eye but it is should be noted that this association was not explored in our study and remains to be investigated. Nonetheless, the considerable delay in presentation for the first affected eye demonstrated in our study highlights the importance of early detection and treatment.

From the patient's perspective, the delay in symptom recognition can be addressed to a certain extent by self-examination. Patients, especially those with an increased risk of developing

For peer review only - http://bmjopen.bmj.com/site/about/guidelines.xhtml

nvAMD, should be educated and made aware of symptoms such as new visual distortion and sudden reduction in vision. This can be achieved by encouraging patients to use suitable spaced self-tests of vision which examine one eye at a time to prevent compensation from the good eye. The standard Amsler test has long been recommended as the standard self-monitoring test but there has been increasing reservation about its utility as a diagnostic tool due to its insufficient reliability and variable sensitivity.[32,33] The advent of more innovative, cost saving technologies may circumvent these issues and make implementation of self-examination on a wider public scale more feasible in the near future.[34,35]

In this study, we chose to investigate patients' awareness of AMD because it is clear that a lack of disease awareness is a common factor for delayed presentation in other eye conditions such as glaucoma, retinal detachment and central retinal artery occlusion.[36,37] The only previous study to investigate AMD awareness in the UK population showed a low awareness (16%).[38] Our study adds to the existing literature by demonstrating that public awareness of AMD is still limited. Our survey shows that awareness of AMD is unacceptably low (37%), especially considering that this condition is the leading cause of blindness in developed countries.[1] The low awareness of AMD is also consistent with the low levels of awareness of AMD in other countries including Australia, Hong Kong, Singapore, Nepal, Bangladesh, China and the United States (range between 5% to 50.5%).[21,38,39–44] It is likely that our findings underestimate the true scale of lack of awareness among the general population because we sampled ophthalmic patients who, by virtue of being surveyed in an eye hospital, are presumably somewhat more attuned to common eye diseases. Our survey also highlights a low awareness of risk factors of AMD (other than age). However, this assessment could be limited by the lack of plausible distractors in the corresponding question which might have increased the respondent's chances of getting a correct answer(s), hence again underestimating the true scale of lack of awareness.

For peer review only - http://bmjopen.bmj.com/site/about/guidelines.xhtml

These findings are important given the severity of the consequences of delayed presentation in AMD and the ready availability of an effective treatment to prevent visual loss. We identified AMD-naive male patients and those with lower education levels to have a particularly low awareness of warning symptoms of AMD, suggesting the need for targeted intervention for these subgroups. As increased awareness can lead patients to seek appropriate medical care, improving awareness would logically lead to better visual prognoses for patients.

There is currently still a need for a unified national awareness campaign on AMD in the UK. A recent report by the Royal National Institute of Blind People highlighted that most initiatives at improving AMD awareness in the UK still operate at a local level.[27] Even then, these efforts often comprised of educational talks targeted at existing patients, rather than raising public awareness. The need for a national campaign has also been recognised by the Macular Society which has made increasing AMD awareness one of the main objectives of its five-year national strategy.[45]

Although some progress has been made since,[46] there is still room for improvement. Current awareness interventions need to be further optimised for a sustained impact. A promising step would be the adoption of the multi-layered approach as adopted by other developed countries.[47] This approach saw the use of a campaign which included a diverse range of activities such as promoting education programmes for patients and primary care, running a national advertising campaign and providing free mobile screening. The end of this focused campaign saw a dramatic increase in AMD awareness and the number of the population requesting fundus examination for symptoms of AMD.[47] The implementation of a similar public health strategy in the UK may achieve similar desirable effects but further research is needed to evaluate the effectiveness of this approach in the UK population. Another important gap highlighted by our study is the underappreciated link between smoking and AMD. This represents a potent novel

For peer review only - http://bmjopen.bmj.com/site/about/guidelines.xhtml
For peer review only

health promotional tool and awareness could be increased by incorporating information in existing campaigns with other smoking related diseases.[48,49]

Lack of awareness and knowledge of correct referral among non-ophthalmologists is also problematic. This may account for the delay in referral demonstrated in our study. A recent national survey revealed that 32% of GPs felt “de-skilled” in diagnosing common eye conditions.[50] The same survey also showed that 38% of GPs felt that eyes are the most difficult part of the body to diagnose. Achieving a better alignment of ophthalmic knowledge between healthcare organisations and professionals will help improve understanding and management of common ophthalmic disorders for those in the front line of eye care.

Conclusion

There is significant delay at every step of the care pathway for patients with nvAMD in south-east Scotland. We also show that awareness and knowledge of AMD are suboptimal. This lack of AMD could account for the long presentation delay of AMD to primary care. This suggests that efforts to educate the public regarding AMD may lead to earlier presentation and hence improved visual outcomes in patients.

Figure Legends

Fig 1. Flow chart depicting the typical care pathway of a patient with neovascular AMD in southeast Scotland. *Based on the recommendations by the Royal College of Ophthalmologists in its 2013 AMD guideline.[14]

Fig 2. Breakdown of the total delay (121.4 days) from symptom onset to treatment for patients with new neovascular AMD in south-east Scotland.

For peer review only - http://bmjopen.bmj.com/site/about/guidelines.xhtml

Acknowledgement

None

Funding

SB was sponsored by the Wellcome Trust Scottish Translational Medicine and Therapeutics Initiative (STMTI) scheme (grant number R42141) during the course of this research.

Competing interests

None declared.

Contributors

BD and SB were involved in conception and design of the study. PS and SG were involved in data acquisition, analysis and interpretation. PS was involved in first draft of manuscript. SG, SB and BD were involved in revising and critically appraising manuscript. PS, SG, BD and SB were involved in final approval for publication. BD and SB are guarantors.

References

1. World Health Organisation. Priority eye diseases. 2017. http://www.who.int/blindness/causes/priority/en/index7.html (accessed 25 Sept 2017).

2. Commissioning contemporary AMD services: a guide for commissioners and clinicians. The Royal College of Ophthalmologists. 2007. http://bmec.swbh.nhs.uk/wp-content/uploads/2013/03/COMMISSIONING-ARMD-SERVICES-JULY-2007.pdf (accessed 25 Sept 2017).

For peer review only - http://bmjopen.bmj.com/site/about/guidelines.xhtml

3. Smith W, Assink J, Klein R, et al. Risk factors for age-related macular degeneration: Pooled findings from three continents. *Ophthalmology* 2001;108:697–704.

4. Minassian DC, Reidy A, Lightstone A, et al. Modelling the prevalence of age-related macular degeneration (2010-2020) in the UK: expected impact of anti-vascular endothelial growth factor (VEGF) therapy. *Br J Ophthalmol* 2011;95:1433–6.

5. Brown DM, Kaiser PK, Michels M, et al. Ranibizumab versus verteporfin for neovascular age-related macular degeneration. *N Engl J Med* 2006;355:1432–44.

6. Brown DM, Michels M, Kaiser PK, et al. Ranibizumab versus verteporfin photodynamic therapy for neovascular age-related macular degeneration: two-year results of the ANCHOR study. *Ophthalmology* 2009;116:57–65.e5.

7. Busbee BG, Ho AC, Brown DM, et al. Twelve-month efficacy and safety of 0.5 mg or 2.0 mg ranibizumab in patients with subfoveal neovascular age-related macular degeneration. *Ophthalmology* 2013;120:1046–56.

8. Heier JS, Brown DM, Chong V, et al. Intravitreal aflibercept (VEGF trap-eye) in wet age-related macular degeneration. *Ophthalmology* 2012;119:2537–48.

9. Rofagha S, Bhisitkul RB, Boyer DS, et al. Seven-year outcomes in ranibizumab-treated patients in ANCHOR, MARINA, and HORIZON: a multicenter cohort study (SEVEN-UP). *Ophthalmology* 2013;120:2292–9.

10. Lim JH, Wickremasinghe SS, Xie J, et al. Delay to treatment and visual outcomes in patients treated with anti-vascular endothelial growth factor for age-related macular degeneration. *Am J Ophthalmol* 2012;153:678–686.

11. Takahashi H, Ohkubo Y, Sato A, et al. Relationship between visual prognosis and delay of intravitreal injection of ranibizumab when treating age-related macular degeneration. *Retina* 2015;35:1331–8.

12. Bonastre J, Le Pen C, Soubrane G, et al. The burden of age-related macular degeneration: results of a cohort study in two French referral centres. *Pharmacoeconomics* 2003;21:181–190.

For peer review only

For peer review only - http://bmjopen.bmj.com/site/about/guidelines.xhtml

13. Hopley C, Salkeld G, Wang JJ, et al. Cost utility of screening and treatment for early age related macular degeneration with zinc and antioxidants. *Br J Ophthalmol* 2004; 88:450–4.

14. Chakravarthy U, Williams M. The AMD Guidelines Group. The Royal College of Ophthalmologists Guidelines on AMD: Executive Summary. *Eye (Lond)* 2013;27:1429–31.

15. Gonzales CR. Enhanced efficacy associated with early treatment of neovascular age-related macular degeneration with pegaptanib sodium: an exploratory analysis. *Retina* 2005;25:815–27.

16. Arias L, Armadá F, Donate J, et al. Delay in treating age-related macular degeneration in Spain is associated with progressive vision loss. *Eye (Lond)* 2009;23:326–33.

17. Garraway WM, Whisnant JP. The changing pattern of hypertension and the declining incidence of stroke. *JAMA* 1987;258:214–7.

18. Worden JK, Solomon LJ, Flynn BS, et al. A community-wide program in breast self-examination training and maintenance. *Prev Med* 1990;19:254–69.

19. Kim YS, Park SS, Bae HJ, et al. Stroke awareness decreases prehospital delay after acute ischemic stroke in Korea. *BMC Neurol* 2011;11:2.

20. Niksic M, Rachet B, Duffy SW, et al. Is cancer survival associated with cancer symptom awareness and barriers to seeking medical help in England? An ecological study. *Br J Cancer* 2016;115:876–86.

21. Livingston PM, McCarty CA, Taylor HR. Knowledge, attitudes, and self care practices associated with age related eye disease in Australia. *Br J Ophthalmol* 1998; 82:780–5.

22. National Statistics. Scottish Index of Multiple Deprivation 2012. http://simd.scotland.gov.uk/publication-2012/ (accessed 25 Sept 2017).

23. Aitken E, Dempster N, Ceresa C, et al. The impact of socioeconomic deprivation on outcomes following renal transplantation in the West of Scotland. *Transplant Proc* 2013;45:2176–83.

24. Low L, Law JP, Hodson J, et al. Impact of socioeconomic deprivation on the development of diabetic retinopathy: a population-based, cross-sectional and longitudinal study over 12 years.

For peer review only

BMJ Open 2015;5:e007290.

25. Cameron JR, Ahmed S, Curry P, et al. Impact of direct electronic optometric referral with ocular imaging to a hospital eye service. *Eye (Lond)* 2009;23:1134–40.

26. Rasmussen A, Brandi S, Fuchs J, et al. Visual outcomes in relation to time to treatment in neovascular age-related macular degeneration. *Acta Ophthalmol* 2015;93:616–20.

27. Royal National Institute of Blind People. Don't lose sight! Don't delay! Perspectives on the wet age-related macular degeneration patient journey. 2013. https://www.rnib.org.uk/sites/default/files/Don%27t%20lose%20sight%20don%27t%20delay%20Campaign%20report.pdf (accessed 25 Sept 2017).

28. Bressler NM. Early detection and treatment of neovascular age-related macular degeneration. *J Am Board Fam Pract* 2002;15:142–52.

29. Chevreaud O, Semoun O, Blanco-Garavito R, et al. Visual acuity at presentation in the second eye versus first eye in patients with exudative age-related macular degeneration. *Eur J Ophthalmol* 2016;26:44–7.

30. Muniraju R, Jayashree R, Sivaprasad S. Outcomes of second eye versus first eye in patients treated with ranibizumab for bilateral neovascular age related macular degeneration. *Invest Ophthalmol Vis Sci* 2013;54:250.

31. Chew J, Broadhead G, Li H, et al. Visual outcomes of ranibizumab treatment in fellow eyes of exudative AMD. *Invest Ophthalmol Vis Sci* 2013;54:3832.

32. Zaidi FH, Cheong-Leen R, Gair EJ, et al. The Amsler chart is of doubtful value in retinal screening for early laser therapy of subretinal membranes. The West London Survey. *Eye (Lond)* 2004;18:503–8.

33. Faes L, Bodmer NS, Bachmann LM, et al. Diagnostic accuracy of the Amsler grid and the preferential hyperacuity perimetry in the screening of patients with age-related macular degeneration: systematic review and meta-analysis. *Eye (Lond)* 2014;28:788–96.

34. Winther C, Frisén L. Self-testing of vision in age-related macula degeneration: a longitudinal

For peer review only

For peer review only - http://bmjopen.bmj.com/site/about/guidelines.xhtml

pilot study using a smartphone-based rarebit test. *J Ophthalmol* 2015;2015:285463.

35. Schwartz R, Loewenstein A. Early detection of age related macular degeneration: current status. *Int J Retina Vitreous* 2015;1:20.

36. Saw S-M, Gazzard G, Friedman D, et al. Awareness of glaucoma, and health beliefs of patients suffering primary acute angle closure. *Br J Ophthalmol* 2003;87:446–9.

37. Uhr JH, Mishra K, Wei C, et al. Awareness and knowledge of emergent ophthalmic disease among patients in an internal medicine clinic. *JAMA Ophthalmol* 2016;134:424–31.

38. AMD Alliance International. Awareness of age-related macular degeneration and associated risk factors. AMD Global Report. 2005. http://www.eyeresearch.org/pdf/AMD_Report_Aug22_1046.pdf (accessed 25 Sept 2017).

39. Lau JTF, Lee V, Fan D, et al. Knowledge about cataract, glaucoma, and age related macular degeneration in the Hong Kong Chinese population. *Br J Ophthalmol* 2002;86:1080–4.

40. Sanjay S, Neo HY, Sangtam T, et al. Survey on the knowledge of age-related macular degeneration and its risk factors among Singapore residents. *Clin Exp Ophthalmol* 2009;37:795–800.

41. Thapa R, Bajimaya S, Paudyal G, et al. Population awareness of diabetic eye disease and age related macular degeneration in Nepal: the Bhaktapur Retina Study. *BMC Ophthalmol* 2015;15:188.

42. Islam FMA, Chakrabarti R, Islam SZ, et al. Factors associated with awareness, attitudes and practices regarding common eye diseases in the general population in a rural district in Bangladesh: The Bangladesh Population-based Diabetes and Eye Study (BPDES). *PLoS One* 2015;10:e0133043.

43. Scott AW, Bressler NM, Ffolkes S, et al. Public attitudes about eye and vision health. *JAMA Ophthalmol* 2016;134:1111–8.

44. Zhang C, Zhang G, Ma N, et al. Awareness of age-related macular degeneration and its risk factors among Beijing residents in China. *Chin Med J (Engl)* 2017;130(2):155–159.

For peer review only

For peer review only - http://bmjopen.bmj.com/site/about/guidelines.xhtml

45. Macular Society. Strategy 2016-2020. 2016. https://www.macularsociety.org/sites/default/files/Macular%20Society%20strategy%202016-2020.pdf (accessed 25 Sept 2017).

46. Royal National Insitute of Blind People. Charity announces new awareness week for macular conditions. 2015. http://www.rnib.org.uk/charity-announces-new-awareness-week-macular-conditions (accessed 25 Sept 2017).

47. Heraghty J, Cummins R. A layered approach to raising public awareness of macular degeneration in Australia. *Am J Public Health* 2012;102:1655–9.

48. Thornton J, Edwards R, Harrison RA, et al. 'Smoke gets in your eyes': A research-informed professional education and advocacy programme. *J Public Health (Oxf)* 2007;29:142–6.

49. Thompson C, Harrison RA, Wilkinson SC, et al. Attitudes of community optometrists to smoking cessation: an untapped opportunity overlooked? *Ophthalmic Physiol Opt* 2007;27:389–93.

50. Optegra Eye Health Care. Vision of Britain Report: an insight into the nation's eye health. 2015. https://www.optegra.com/vob-report.pdf (accessed 25 Sept 2017).

For peer review only

For peer review only - http://bmjopen.bmj.com/site/about/guidelines.xhtml

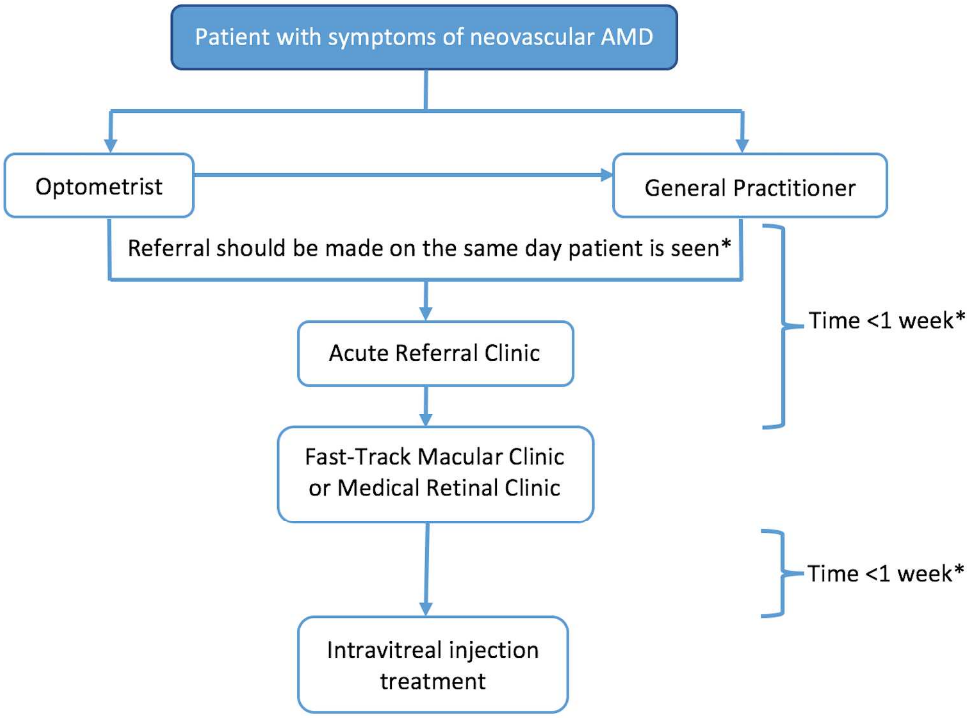

Fig 1. Flow chart depicting the typical care pathway of a patient with neovascular AMD in south-east Scotland. *Based on the recommendations by the Royal College of Ophthalmologists in its 2013 AMD guideline.[14]

For peer review only

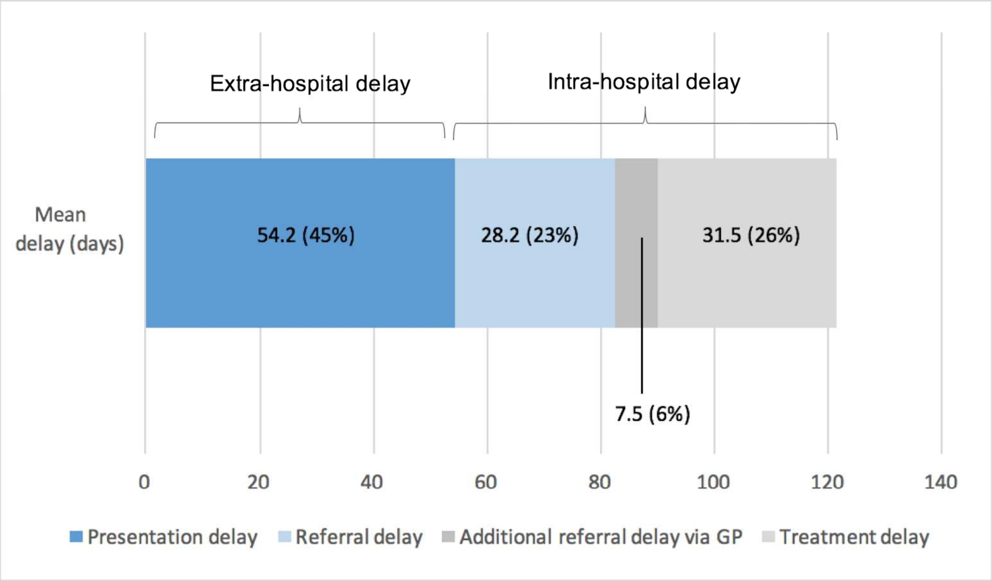

Fig 2. Breakdown of the total delay (121.4 days) from symptom onset to treatment for patients with new neovascular AMD in south-east Scotland.

review only

Appendix 1: AMD Awareness Questionnaire.

No: Postcode:

AMD Awareness Questionnaire

Please circle the correct option

1. **Sex:** Male Female

2. **Age (years):**

3. **Educational Status:**
   1) Completed Primary School
   2) Completed Secondary School
   3) College Qualification
   4) University Degree
   5) Prefer not to say

4. **Employment status:**
   1) Unemployed
   2) Full time
   3) Part time
   4) Retired
   5) Other (specify):
   6) Prefer not to say

5. **Prior to now, have you ever been told you have Age Related Macular Degeneration (AMD)?**
   1) Yes
   2) No

IF 'Yes', MOVE ON TO QUESTION 7

6. **If no, have you ever heard of Age Related Macular Degeneration (AMD)?**
   1) Yes
   2) N

For peer review only

7. **If you have heard of AMD, can you describe the condition and its symptoms?**

____

____

8. **Regardless of whether or not you are familiar with AMD, which of the following factors do you think increases the risk of developing AMD? (select all that apply)**
   1) Smoking
   2) Vitamin deficiency
   3) Age
   4) Unprotected exposure to sunlight
   5) Genetics
   6) Sex

9. **Do you currently smoke?**
   1) Yes
   2) No

10. **Is AMD a treatable condition?**
    1) Yes
    2) No

11. **If yes, do you know what treatments are available?**

____

____

12. **If you were worried about your eyesight, where would you go for advice**?
    1) Ophthalmologist
    2) GP
    3) Pharmacist
    4) Optician
    5) Other (specify):

For peer review only

Appendix 2: Power calculation for AMD awareness survey sample size.

Sample Size:X-Sectional, Cohort, & Randomized Clinical Trials

Two-sided significance level(1-alpha):	95
Power(1-beta, % chance of detecting):	80
Ratio of sample size, Unexposed/Exposed:	1
Percent of Exposed with Outcome:	25
Odds Ratio:	6.3
Risk/Prevalence Ratio:	5
Risk/Prevalence difference:	20

	Kelsey	Fleiss	Fleiss with CC
Sample Size - Exposed	51	49	59
Sample Size-Nonexposed	51	49	59
Total sample size:	102	98	118

References

Kelsey et al., Methods in Observational Epidemiology 2nd Edition, Table 12-15
Fleiss, Statistical Methods for Rates and Proportions, formulas 3.18 &3.19
CC = continuity correction

Based on a global survey investigating AMD awareness by AMD Alliance International, the level of awareness in the UK was 16% in 2005.[38] Allowing for increase in awareness over time (demonstrated by studies in other countries), hence assuming a slightly higher level of awareness (~25%) in south-east Scotland, we would have a power of 80% to detect this with a total sample size of 118 patients.

Appendix 3: Table comparing summary demographic data for included vs excluded case notes.

Patient demographics	Analysed (*n* = 195)	Excluded (*n* = 120)
Sex (% female)	61.5	58.3
Mean age (years)	77.7	78.4
Percentage of patients presenting with first affected eye	95.9%	97.5%

Appendix 4: Table showing breakdown of case notes excluded from study.

**Total case notes identified**	**315**
Co-existence of ocular comorbidities that give rise to choroidal neovascularization	23
Symptom duration not recorded	76
Lost to follow-up	21
**Case notes included in study**	**195**

STROBE 2007 (v4) Statement—Checklist of items that should be included in reports of *cross-sectional studies*

Section/Topic	Item #	Recommendation	Reported on page #
**Title and abstract**	1	(*a*) Indicate the study's design with a commonly used term in the title or the abstract	1
		(*b*) Provide in the abstract an informative and balanced summary of what was done and what was found	2
**Introduction**			
Background/rationale	2	Explain the scientific background and rationale for the investigation being reported	4-5
Objectives	3	State specific objectives, including any prespecified hypotheses	6
**Methods**			
Study design	4	Present key elements of study design early in the paper	6
Setting	5	Describe the setting, locations, and relevant dates, including periods of recruitment, exposure, follow-up, and data collection	6
Participants	6	(*a*) Give the eligibility criteria, and the sources and methods of selection of participants	6
Variables	7	Clearly define all outcomes, exposures, predictors, potential confounders, and effect modifiers. Give diagnostic criteria, if applicable	6
Data sources/ measurement	8*	For each variable of interest, give sources of data and details of methods of assessment (measurement). Describe comparability of assessment methods if there is more than one group	6
Bias	9	Describe any efforts to address potential sources of bias	6-7
Study size	10	Explain how the study size was arrived at	6-7
Quantitative variables	11	Explain how quantitative variables were handled in the analyses. If applicable, describe which groupings were chosen and why	6-7
Statistical methods	12	(*a*) Describe all statistical methods, including those used to control for confounding	6-7
		(*b*) Describe any methods used to examine subgroups and interactions	6-7
		(*c*) Explain how missing data were addressed	6-7
		(*d*) If applicable, describe analytical methods taking account of sampling strategy	N/A
		(*e*) Describe any sensitivity analyses	6-7
**Results**			

For peer review only

Participants	13*	(a) Report numbers of individuals at each stage of study—eg numbers potentially eligible, examined for eligibility, confirmed eligible, included in the study, completing follow-up, and analysed	6, 8
		(b) Give reasons for non-participation at each stage	6, 8
		(c) Consider use of a flow diagram	N/A
Descriptive data	14*	(a) Give characteristics of study participants (eg demographic, clinical, social) and information on exposures and potential confounders	7, 9
		(b) Indicate number of participants with missing data for each variable of interest	6, 8
Outcome data	15*	Report numbers of outcome events or summary measures	7-10
Main results	16	(*a*) Give unadjusted estimates and, if applicable, confounder-adjusted estimates and their precision (eg, 95% confidence interval). Make clear which confounders were adjusted for and why they were included	7-10
		(*b*) Report category boundaries when continuous variables were categorized	7-10
		(*c*) If relevant, consider translating estimates of relative risk into absolute risk for a meaningful time period	N/A
Other analyses	17	Report other analyses done—eg analyses of subgroups and interactions, and sensitivity analyses	7-10
**Discussion**			
Key results	18	Summarise key results with reference to study objectives	11, 13
Limitations	19	Discuss limitations of the study, taking into account sources of potential bias or imprecision. Discuss both direction and magnitude of any potential bias	13
Interpretation	20	Give a cautious overall interpretation of results considering objectives, limitations, multiplicity of analyses, results from similar studies, and other relevant evidence	11-14
Generalisability	21	Discuss the generalisability (external validity) of the study results	11-14
**Other information**			
Funding	22	Give the source of funding and the role of the funders for the present study and, if applicable, for the original study on which the present article is based	15

*Give information separately for cases and controls in case-control studies and, if applicable, for exposed and unexposed groups in cohort and cross-sectional studies.

Note: An Explanation and Elaboration article discusses each checklist item and gives methodological background and published examples of transparent reporting. The STROBE checklist is best used in conjunction with this article (freely available on the Web sites of PLoS Medicine at http://www.plosmedicine.org/, Annals of Internal Medicine at http://www.annals.org/, and Epidemiology at http://www.epidem.com/). Information on the STROBE Initiative is available at www.strobe-statement.org.

For peer review only
